# A privacy-preserving approach for cloud-based protein fold recognition

**DOI:** 10.1016/j.patter.2024.101023

**Published:** 2024-07-19

**Authors:** Ali Burak Ünal, Nico Pfeifer, Mete Akgün

**Affiliations:** 1 Medical Data Privacy and Privacy Preserving Machine Learning (MDPPML), Department of Computer Science, University of Tübingen, 72076 Tübingen, Germany; 2 Methods in Medical Informatics, Department of Computer Science, University of Tübingen, 72076 Tübingen, Germany; 3 Institute for Bioinformatics and Medical Informatics (IBMI), Department of Computer Science, University of Tübingen, 72076 Tübingen, Germany

**Keywords:** protein fold recognition, machine learning as a service, recurrent kernel networks, data privacy, multi-party computation, cloud-based machine learning, privacy preserving machine learning

## Abstract

The complexity and cost of training machine learning models have made cloud-based machine learning as a service (MLaaS) attractive for businesses and researchers. MLaaS eliminates the need for in-house expertise by providing pre-built models and infrastructure. However, it raises data privacy and model security concerns, especially in medical fields like protein fold recognition. We propose a secure three-party computation-based MLaaS solution for privacy-preserving protein fold recognition, protecting both sequence and model privacy. Our efficient private building blocks enable complex operations privately, including addition, multiplication, multiplexer with a different methodology, most-significant bit, modulus conversion, and exact exponential operations. We demonstrate our privacy-preserving recurrent kernel network (RKN) solution, showing that it matches the performance of non-private models. Our scalability analysis indicates linear scalability with RKN parameters, making it viable for real-world deployment. This solution holds promise for converting other medical domain machine learning algorithms to privacy-preserving MLaaS using our building blocks.

## Highlights

• We propose private machine learning as a service for protein fold recognition
• We combine recurrent kernel networks and multi-party computation
• Our approach computes the same result as plaintext RKN without compromising privacy
• We show its linear scalability to the parameters of recurrent kernel networks

## The bigger picture

In the era of cloud-based machine learning, privacy concerns, especially in medicine, are critical. Protecting the privacy of medical data is essential for maintaining patient trust and complying with regulations. Recognizing protein folds is vital for understanding diseases and developing treatments, but it currently lacks a privacy-preserving solution. We present an approach that secures this process, allowing the use of advanced models without compromising data or model privacy. By maintaining high performance while ensuring privacy, our scalable and efficient solution demonstrates the practicality of secure cloud-based machine learning in healthcare. This work highlights the urgent need for privacy-conscious cloud-based machine learning and aims to inspire further advancements, emphasizing the importance of data privacy in medical applications.

## Teaser

This work proposes the first privacy-preserving machine-learning-as-a-service approach for protein fold recognition tasks. It utilizes multi-party computation to perform inference on the query sequence via pre-trained recurrent neural networks. The authors design and implement several efficient multi-party computation building blocks to address the required operations in recurrent kernel networks. They demonstrate its correctness on the Structural Classification of Proteins dataset and the scalability of the solution to various parameters on a synthetic dataset.

## Introduction

Machine learning as a service (MLaaS) has become so popular recently due to its efficiency and practicality in various domains. With the increased complexity and cost of training machine learning algorithms, it has become challenging for businesses and researchers to develop and deploy these models in-house, as such an action would require considerable expertise and computational power. Cloud-based MLaaS solutions provide access to pre-trained models, avoiding the need for expensive hardware and software investments and reducing the time and resources needed to develop a model from scratch. Thanks to its efficiency and practicality, MLaaS has been successfully applied to various domains,^1^^,^^2^^,^^3^ including the medical domain.

One specific problem in the medical domain is the protein fold recognition task. The structure of a protein is one of the factors determining its functionality.^4^^,^^5^ The shape of a protein, for instance, affects its ability to bind to other proteins.^6^^,^^7^ One of the steps toward modeling the structure of a protein is to determine the folds of a protein by comparing the given protein sequence to the sequences of proteins with known structures.^8^ By this approach, one can predict the structure of a protein and assess its functionality to some extent. As an illustrative example of why such information is important, consider a patient with mutations in several of their genes. Determining whether these mutations affect the structure of the proteins that are synthesized based on these genes can help physicians select the correct treatment for the patient, leading to literally a life-or-death decision. In the literature, there are several different approaches proposed for protein fold recognition such as DeepSVM-Fold,^9^ DeepMSA,^10^ AlphaFold,^11^^,^^12^ ESMFold,^13^ and recurrent kernel networks (RKNs).^14^ Among these approaches, even though AlphaFold and ESMFold are MLaaS solutions for protein fold recognition, they do not utilize any privacy-enhancing technique and, as a natural outcome, protect the sensitive data in the query sequences. To the best of our knowledge, the submitted query sequence to AlphaFold and ESMFold can be accessible in plaintext by the server. This access to the protein sequences poses a significant challenge. The sequences contain sensitive information about the individual from whom they were derived, such as genetic predispositions to certain diseases, and this information about the corresponding individual can be compromised by the server owner. In addition to the input sequence’s privacy, the model’s privacy can be an issue if the model is proprietary and the owner of the model does not have enough computational power to provide MLaaS. To allow others to benefit from the model, the owner has to outsource the model to a third party. However, outsourcing the model in plaintext risks the intellectual property of the model, and the third party could use the model in additional scenarios without the knowledge of the developer of the model. In summary, even though there exist some MLaaS protein fold recognition approaches, to the best of our knowledge, there exists no privacy-preserving protein fold recognition approach proposed in the literature.

To address the need to protect the privacy of the protein sequences and the model, a natural path is to integrate a privacy-enhancing technique into the process. Various privacy-enhancing techniques have been proposed in the literature to protect sensitive data during such operations. One of these techniques is differential privacy (DP). It introduces noise to a phase or several phases of machine learning training and/or testing to protect the privacy of the data and the model.^15^^,^^16^ However, DP can significantly reduce the accuracy of a model since its main mechanism to provide privacy is to add noise to the data/model parameters. Another technique is homomorphic encryption (HE), where all the computations are performed on encrypted data.^17^^,^^18^^,^^19^^,^^20^ The computations on the homomorphically encrypted data do not reveal any information about the underlying data thanks to their encrypted nature. However, the limited operations offered by HE and its computational expensiveness make it impractical for real-world applications. Secure multi-party computation (MPC), however, addresses the missing points of HE and the fundamental privacy requirements. The data and the model parameters are shared among several parties in such a way that none of the parties can learn about the data and/or the model parameters on their own. Then, these parties perform the desired computation privately. To address various machine learning algorithms, there are several MPC frameworks in the literature,^21^^,^^22^^,^^23^^,^^24^^,^^25^^,^^26^^,^^27^ some of which also utilize HE.^28^^,^^29^ Their focus is, however, to address mostly convolutional neural network (CNN) models in a privacy-preserving way, and the building blocks of these MPC frameworks are customized to perform CNN operations efficiently.

Compared to the large architecture and complexity of AlphaFold and ESMFold, RKNs^14^ have a more privacy-friendly deep learning architecture. Chen et al.^14^ gave a kernel perspective of recurrent neural networks (RNNs) by showing that the computation of the specific construction of RNNs, which they call RKNs, mimics the substring kernel allowing mismatches and the local alignment kernel, which are widely used on sequence data.^30^^,^^31^^,^^32^ In RKNs, small motifs called anchor points are used as templates to measure similarities among sequences. By traversing every character of the sequence, the overall search for a mapping of anchor points is performed, and the final mapping of the sequence is computed by multiplying the initial mapping and the inverse square root of the gram matrix of the anchor points. Then, the classifier layer gives the prediction score of the sequence. Thanks to the combination of a well-designed kernel formulation and parameter optimization through backpropagation, RKNs outperform the traditional substring kernel and the local alignment kernel, as well as long short-term memories (LSTMs).^33^

**Figure 1:**
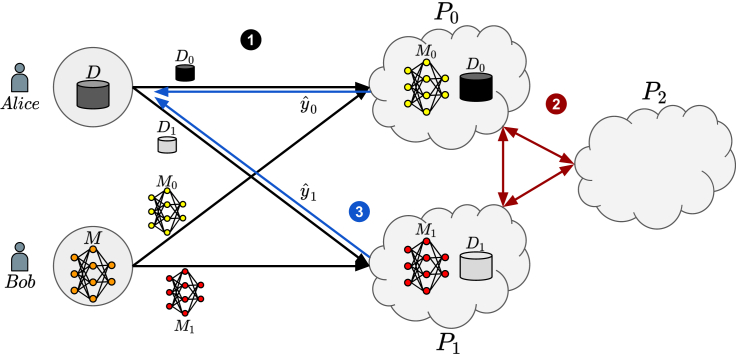
The overview of our privacy-preserving RKN as a service via MPC (1) At first, the data owner, i.e., Alice, secret shares her data and sends the shares to the proxies. Similarly, the model owner, i.e., Bob, does the same with the parameters of the model. (2) Then, by using the outsourced model and the data, the proxies, $P_{0}$ and $P_{1}$, perform the operations required for the inference of the data on the model with the help of $P_{2}$, which is the helper. (3) Finally, the proxies send the shares of the prediction of the given data to the data owner.

Considering the merits of RKNs and well-combined privacy and computational efficiency features of MPC, in this paper, we address the necessity of private and secure MLaaS for protein fold recognition by proposing privacy-preserving RKN as a service using MPC. In our solution, an overview of which is given in Figure 1, we perform protein fold recognition on a given sequence without sacrificing the privacy of the sequence or the model. More specifically, both the input sequence and model parameters are secret shared to computing parties so that neither the model owner nor the sequence owner has to sacrifice their confidential information for the sake of the inference. The realization of these operations accurately via existing MPC frameworks, however, is a challenging task, if not impossible. Many MPC frameworks in the literature are designed for CNNs, and it is difficult to adapt them to new problems due to a lack of documentation and code flexibility. While we benefit from existing basic MPC operations such as addition and multiplication to perform the privacy-preserving RKN as a service, we design and implement several highly efficient MPC building blocks to perform the classification of the given protein sequence on the outsourced pre-trained RKN model without compromising the privacy of the sequence data or the model parameters. We call the resulting MPC framework CECILIA. As a summary, our contributions can be listed as follows.

Privacy-preserving RKN as a service: we propose the privacy-preserving MLaaS protein fold recognition approach based on RKNs. The performance of RKNs, thanks to their well-established kernel method basis and backpropagation—allowing deep neural network yet privacy-friendly architecture—made RKN the best choice for this task.
Efficient MPC building blocks: considering the lack of comprehensive documentation, flexibility, and user-friendly interfaces of the existing MPC frameworks, we designed and implemented highly efficient MPC primitives, resulting in an MPC framework, CECILIA, to address these limitations. These primitives include the conversion of shares from the $2^{n-1}$ ring to the $2^{n}$ ring, known as modulus conversion (MOC), the computation of the most significant bit (MSB) of secret-shared values, the randomized encoding (RE)-based secret-shared multiplexer (MUX) to select, and the secret-shared accurate exponentiation (EXP).

## Results

### Overview of RKN as a service

#### Setup

The setup starts with outsourcing. In the outsourcing of the model parameters, which are the anchor point matrix, the linear classifier weights, and the inverse square root of the gram matrices of the anchor points, the model owner secret shares them and sends these shares to the proxies, which are two of three computing parties interacting with the users, such that each proxy has a single share of each parameter. To outsource the test samples, the data owner proceeds similarly after using one-hot encoding to convert a sequence into a vector of numbers. It divides this vector into two shares and sends them to the proxies. Besides outsourcing, the proxies agree on a common seed to generate common randoms.

#### Private inference

**Figure 2:**
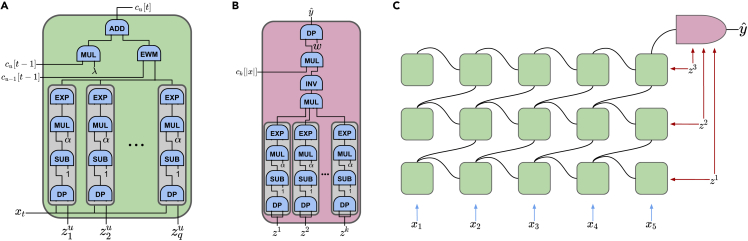
The architecture of RKN and internal computations of its layers (A and B) The arithmetic circuits of (A) a single neuron of RKN at position *t* and *k*-mer level *u* and (B) the linear classifier layer of RKN after the last position of the input sequence *x* are depicted. $z_{i}^{j}$ represents the *i*-th character of the *j*-th anchor point, and $c_{i}\left[j\right]$ represents the initial mapping of the sequence up to its *j*-th character into a *q*-dimensional vector based on anchor points of length *i* where i∈{1,…,k}, j∈{1,…,s}, and *q* is the number of anchor points. (C) For k=3 and |x|=5, RKN is shown where the green nodes are the single neurons and the pink one is the linear classifier.

After the setup, we use the building blocks for private inference on a pre-trained RKN as MLaaS, whose internal computations are given in Figure 2. Let t∈{1,…,|x|} be the index of the characters in the sequence *x*. First, the proxies compute $b_{j}\left[t\right]$, which is the similarity of the one-hot-encoded *t*-th character of the sequence to the *j*-th character of each anchor point for j∈{1,…,k}. As shown in the gray boxes in Figure 2A, this calculation involves the dot product of two secret-shared vectors, the subtraction of a plaintext scalar value from a secret-shared value, the multiplication of a plaintext scalar value by a secret-shared value, and the exponential of a known base raised to the power of a secret-shared value. Once the similarity computation is complete, the proxies proceed with the private element-wise product between $b_{j}\left[t\right]$ and $c_{j-1}\left[t-1\right]$, which is the initial mapping of the sequence up to the (t−1)-th character based on the anchor points of length (j−1). Then, the proxies add the result of the element-wise product with the downgraded $c_{j}\left[t-1\right]$ with a plaintext scalar value λ. At the end of this computation, the proxies obtain the secret-shared initial mapping of the sequence $c_{j}\left[t\right]$ up to the *t*-th character to *q*-dimensional space based on each anchor point of length j∈{1,…,k}.

After computing the full initial mapping of the sequence, that is, $c_{k}\left[\left|x\right|\right]$, the proxies multiply the inverse square root of the gram matrices $K_{Z_{k}Z_{k}}^{-1/2}$ by the corresponding initial mapping vectors of the sequence. Afterward, the proxies compute the private dot product of two secret-shared vectors, which are the weights of the classifier and the mapping of the sequence. In the end, they obtain the secret-shared prediction of the given sequence. These shares can then be sent back to the owner of the data, enabling the reconstruction of the prediction.

### Dataset

To perform the protein fold recognition on RKN as a service, we utilized Structural Classification of Proteins (SCOP) v.1.67,^34^ which was also used by Chen et al.^14^ It contains 85-fold recognition tasks with positive or negative labels and protein sequences of varying lengths. The comparison of the predictions of our privacy-preserving RKN as a service and plaintext RKN on SCOP demonstrates the correctness of our approach. We also analyze the scalability of it to various parameters of RKN. For this purpose, we use a synthetic dataset.

### Experimental setup

We conducted the experiments on a dedicated server with an Intel Xeon Gold 6140 CPU running at 2.30 GHz, equipped with 256 GB of memory, and running Ubuntu. We ran our experiments on local area network (LAN) and wide area network (WAN) settings. To simulate the WAN setting, we set the average round-trip time of the local host to 20 ms. We represent numbers with 64 bits whose least significant 20 bits are used for the fractional part.

### Experimental evaluation

#### Correctness analysis

We selected tasks of SCOP and trained an RKN on them using the same parameter setting as Chen et al.,^14^ that is, q=128 and k=10. Then, we outsource the parameters of the model to the proxies, which are the anchor points, biases, inverse square root of the anchor points, and linear classifier. All computing parties are connected via LAN. To perform the classification of the protein sequences in the test set of the selected task, we outsource those sequences to the proxies as well. Once the model and the test samples are outsourced, the proxies perform the sequence of required operations and obtain the results in secret-shared form. Then, they return the shares of these results, and we reconstruct them as plaintext. When we compared the predictions of our privacy-preserving RKN as a service with the predictions of the plaintext RKN model, the largest absolute difference between the corresponding predictions is less than $2\times 10^{-5}$, which is an expected difference with fixed-point arithmetic. Such close predictions suggest that our MPC-based RKN as a service can yield the correct results without compromising the privacy of the protein sequence or the model parameters. To give a better perspective on how an inference in real-life deployment would look considering that these sequences are real sequences, we analyze the runtime of performing inference on these sequences. On average, an inference of a query sequence via RKN as a service takes 3.9 s, while the corresponding plaintext inference takes around $3.6\times 10^{-5}$ s. Considering the required communication between computing parties, the complexity of operations, and the cumbersome nature of getting the required permissions to use the data in plaintext, if possible at all, it is fair to state that our runtime is acceptable. In the case of extending the capability of this service to respond to multiple requests at the same time via parallelization, the total amount of time required to perform inference on a set of query sequences can be further reduced.

#### Execution time analysis

**Figure 3:**
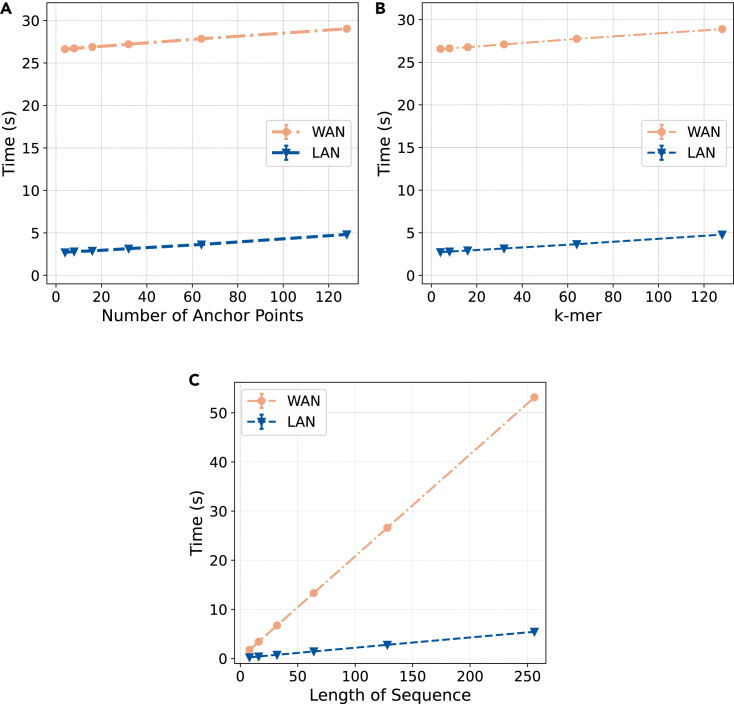
The results of the execution time analysis of our RKN as a service The results of the execution time analysis of our RKN as a service on both WAN and LAN settings for varying (A) numbers of anchor points for a fixed k-mer length and sequence length, (B) lengths of k-mers for a fixed number of anchor points and sequence length, and (C) lengths of sequences for a fixed number of anchor points and k-mer length.

We examined the effects of the parameters of the RKN, namely the number of anchor points, the length of the k-mers, and the sequence length, on the execution time of our RKN as a service on both LAN and WAN. To do this, we curated datasets of synthetic protein sequences, focusing on the runtime of the classification rather than its correctness. For the analysis of the number of anchor points and the length of the k-mers, we used a dataset of fixed-length sequences, specifically 128 amino acids for each sequence. To observe the impact of sequence length on execution time, we created a dataset with varying sequence lengths. In our analyses, we varied the parameter of interest while keeping the others fixed for better observation. When analyzing the impact of the number of anchor points on execution time using fixed-length protein sequences, we set the length of k-mers to 8. Similarly, we fixed the number of anchor points to 8 to analyze the impact of the length of k-mers on execution time. To observe the impact of the sequence length, we fixed both the number of anchor points and the length of k-mers to 8. We repeated each experiment 5 times and report their results to have a robust and fair evaluation. Figure 3 summarizes the results of these experiments and illustrates the linear trend in the execution time of the privacy-preserving RKN as a service for different parameters on both LAN and WAN settings.

## Discussion

In this study, we introduce the privacy-preserving protein fold recognition in MLaaS by proposing privacy-preserving RKN as a service using MPC. We address the privacy issue in protein fold recognition, which has been overlooked in the literature so far. None of the deployed MLaaS protein fold recognition algorithms have considered the security of the query protein sequence. Thanks to our MPC-based solution, MLaaS protein fold recognition without revealing the query sequence is possible for all sorts of entities and individuals, for some of whom it could have not been possible due to privacy concerns and regulations. A hospital, for instance, could not benefit from a non-private MLaaS protein fold recognition algorithm due to data protection and data security regulations. The hospital is not allowed to send the data of patients outside in plaintext, considering that such an action would compromise the sensitive information of patients to third parties. The structure would be important in scenarios where it is expected that a change in the protein sequence of the patient will lead to a change in the protein structure, but this means that the information can be very sensitive if the change in the protein sequence leads to a change in the structure, which leads to a loss or gain of function of the protein and, potentially, to a disease.^34^^,^^35^^,^^36^^,^^37^ Therefore, unless the query protein sequence is kept private during the whole operation, such protein fold recognition services have no use for entities and individuals who have privacy concerns. In our solution, we preserve the privacy of the query sequence during the whole inference process, and none of the sensitive information is revealed to the computing parties. This will allow the utilization of our MLaaS protein fold recognition algorithm to be used by anyone with a protein sequence.

RKN as a service utilizes MPC to ensure that the sensitive information of the query protein sequence is not compromised. It also addresses the protection of the model parameter privacy, which is especially important when the model is proprietary and outsourced to third parties to provide MLaaS protein fold recognition. In RKN as a service, both the query protein sequence and the model parameters are secret shared to two proxies, allowing them to hold only a single share that does not reveal anything about the original value. They then use randomization to mask the data and perform the required operations with the help of the third party. In the end, they obtain the result in secret-shared form, meaning that they cannot learn the prediction of the RKN model for the given protein sequence. Only the owner can recover the prediction in plaintext after receiving these shares back from the computing parties.

The choice of MPC to provide security and privacy allows us to perform the protein fold recognition task efficiently compared to HE and accurately compared to DP. While providing complete security, MPC is more efficient than HE, allowing us to realize the required operations for RKN as a service in a feasible time frame, as stated in the experimental evaluation section. This makes our MPC-based RKN-as-a-service solution favorable over possible HE-based solutions when the efficiency of the solution is a key criterion. Compared to DP, our MPC-based solution provides more accurate results thanks to its exact computation. Due to the noise addition in DP, the result differs from the result that one would obtain in plaintext protein fold recognition using the same model and the query sequence. Moreover, adding a sufficient amount of noise to the query sequence is not possible when one-hot encoding is used without destroying the one-hot encoding completely. Since the values of the encoding are known to be either 0 or 1, a small amount of noise would not be able to hide these values. A large amount noise, on the other hand, would completely destroy the one-hot encoding, leading to a significant performance loss of the model. This issue can be resolved by making the noisy model publicly available such that the users can use this model locally. However, this contradicts the idea of MLaaS solutions. In summary, when the accuracy of the deployed model is prioritized, our MPC-based solution stands out among possible DP-based solutions.

Our analyses demonstrate the efficiency and accuracy of RKN as a service. In our experiments with SCOP, we have shown that the privacy-preserving RKN as a service is capable of making the same predictions as its plaintext, non-private counterpart. This proves an improvement in the existing methodology, as we do not sacrifice the accuracy of the protein fold recognition model for the sake of privacy, as would be the case for approaches based on ϵ− DP. Instead, we maintain the model’s performance while ensuring the privacy of both the input sequence and the model parameters. Moreover, our experiments with synthetic data have shown that the privacy-preserving RKN as a service scales linearly with the number of anchor points, the length of k-mers, and the length of the input sequence, as shown in Figure 3. The privacy-preserving RKN as a service requires a reasonable amount of time to perform inference on the input sequence, suggesting that it can be deployed in real-life scenarios to protect sensitive information in the input sequence and the model. A detailed analysis of communication round complexity of the MPC building blocks can be found in Table S1.

### Limitations of the study

While we enable the privacy-preserving inference on a pre-trained RKN model that is outsourced to the proxies, we do not provide the users with the option of fine-tuning this model for their specific tasks. To achieve a meaningful improvement in the model performance via fine-tuning, the anchor points acting as a template to measure the similarities between sequences need to be fine-tuned, leading to training the network again. However, due to the complexity and uniqueness of RKN operations, this is infeasible using the existing MPC protocols. For instance, one has to be able to compute the inverse square root of the kernel matrix of anchor points, which has not been addressed in the literature. Such a limitation of our solution could be disadvantageous when a user requires a slightly specialized model for their needs and using the existing model could lead to wrong results. One *ad hoc* solution to this issue is to retrain or fine-tune the model using a dataset containing sequences similar to the ones that the user has. Afterward, this newly trained/fine-tuned model can be outsourced to the computing servers, and the user and other users with similar sequences can utilize this model to perform protein fold recognition. Even though such an *ad hoc* solution may solve the problem in some cases, there are cases where such a dataset may not be available for the model owner to retrain/fine-tune the existing model. Therefore, an efficient solution to fine-tuning the existing model for individual users remains an open research problem. Furthermore, we consider only the semi-honest adversary, in other words, the honest-but-curious threat model. Extending our current solution to the malicious adversary is a relatively challenging task. One of the main reasons is the underlying 2-out-of-2 additive secret sharing. In this secret-sharing scheme, a secret value is secret shared using two unique values, making designing maliciously secure MPC protocols highly challenging. First and most importantly, this system cannot provide security in the case of two malicious proxies, that is, the computing parties with the secret shares. They can easily reconstruct the secret value when they are both malicious. In the case of having a malicious proxy and a malicious helper, they can also retrieve the secret value during the computation. For instance, the helper provides random values, which are called multiplication triples, to the proxies during the multiplication operation. In the process, the proxies reconstruct the secret value masked using these random values. Knowing these random values and the masked secret value allows the malicious adversary corrupting the helper and a proxy to obtain the secret value. Therefore, the current system cannot handle a malicious majority. This means that there could be at most a single malicious adversary in the system. Even though a single malicious adversary cannot retrieve the secret value, it can still lead to an incorrect result. A general approach to address this issue in the presence of a malicious adversary in the honest-majority setting in the 2-out-of-2 additive secret-sharing scheme is to perform extra side operations to verify the correctness of the result. This approach is called verifiable computing, consisting of different techniques such as cut and choose.^38^ However, this would lead to too many extra computations and, naturally, significant overhead in the runtime. A possible future research direction would be to perform the privacy-preserving RKN as a service in the presence of a malicious adversary without introducing too much extra computation into the current solution.

Overall, our privacy-preserving RKN as a service allows entities and individuals to use an MLaaS protein fold recognition algorithm without compromising the sensitive information of the input sequence in the presence of a semi-honest adversary. Such privacy protection is not just for the users of this model but also for the owner of the model. The owner of the RKN model does not have to reveal the model to deploy the model as an MLaaS either.

## Experimental procedures

### Resource availability

#### Lead contact

Further information and requests for resources should be directed to the lead contact, Ali Burak Ünal (ali-burak.unal@uni-tuebingen.de).

#### Materials availability

No new biological materials were generated by this study.

#### Data and code availability

This study uses previously published datasets. We refer to the reader to Murzin et al.^39^ to obtain the SCOP dataset and Chen et al.^14^ for data preparation steps. Considering that the purpose of the synthetic dataset is to analyze the runtime, not the correctness, we generated the synthetic dataset randomly at runtime. The source code of our privacy-preserving RKN-as-a-service solution is available at Github (https://github.com/mdppml/RKN-as-a-Service) and has been archived at Zenodo.^40^

### MPC

MPC is one of the privacy-enhancing techniques based on cryptography where the owner of a secret input secret shares it to two or more computing parties. These computing parties collaborate to collectively compute a function without revealing any participant’s complete input. The secret-sharing mechanism ensures that sensitive information remains private, allowing for joint computations while preserving the confidentiality of individual inputs. In 2-out-of-2 additive secret sharing, for instance, the participants divide their inputs into two values such that the individual shares do not reveal anything about the other share and their summation over a specific ring gives the secret input. MPC is especially useful in scenarios where multiple parties need to collaborate on data analysis, and the computation is outsourced to external entities while maintaining the privacy of the original inputs.

### Notations

We use 2-out-of-2 additive secret sharing over three different rings$,Z_{L}$, $Z_{K}$, and $Z_{V}$, where $L=2^{n}$, $K=2^{n-1}$, V=67, and n=64. We denote two shares of *x* over $Z_{L}$, $Z_{K}$, and $Z_{V}$ by (${\left\langle x\right\rangle }_{0}$, ${\left\langle x\right\rangle }_{1}$), (${\left\langle x\right\rangle }_{0}^{K}$, ${\left\langle x\right\rangle }_{1}^{K}$), and (${\left\langle x\right\rangle }_{0}^{V}$, ${\left\langle x\right\rangle }_{1}^{V}$), respectively. If a value *x* is shared over the ring $Z_{V}$, then every bit of *x* is additively shared in $Z_{V}$. This means *x* is shared as a vector of *n* shares, where each share takes a value between 0 and (V−1). We also use Boolean sharing of a single bit denoted by (${\left\langle x\right\rangle }_{0}^{B}$, ${\left\langle x\right\rangle }_{1}^{B}$).

### MPC building blocks

The building blocks of our solutions are based on three computing parties and 2-out-of-2 additive secret sharing. In the resulting MPC framework, which we call CECILIA, two of these parties, $P_{0}$ and $P_{1}$, are called proxies, and the external entities such as the model owner and the data sources interact with them. The third one, $P_{2}$, is the helper party, helping the proxies compute the desired function without breaking the privacy. It provides the proxies with the shares of purposefully designed values. It also performs calculations on the data masked by the proxies and returns the share of the results of these calculations. In our solution, we use fixed-point arithmetic to be able to represent and work on real numbers. A detailed explanation of the number format as well as the algorithms of the complex building blocks can be found in the supplemental information.

#### Addition

The proxies add the shares of two secret-shared values they have to obtain the share of the addition of these values without any communication or privacy leakage.

#### Multiplication

The multiplication operation, which we adapted from SecureML, uses the pre-computed multiplication triples^41^ and requires truncation because of the special number format. For more details, please refer to Wagh et al.^22^ and Mohassel and Zhang.^23^

#### Modulo conversion

We offer the functionality MOC converting shares over $Z_{K}$ to fresh shares over $Z_{L}$, where L=2K. Even though other frameworks in the literature have functions with a similar name, none of them perform this specific MOC. SecureNN,^22^ for instance, offers *ShareConvert* to convert the shares from $2^{64}$ to $2^{64}-1$. Assuming that $P_{0}$ and $P_{1}$ have the shares ${\left\langle x\right\rangle }_{0}^{K}$ and ${\left\langle x\right\rangle }_{1}^{K}$, respectively, the first step for $P_{0}$ and $P_{1}$ is to mask their shares by using the shares of the random value $r\in Z_{K}$ sent by $P_{2}$. Afterward, they reconstruct $\left(x+r\right)\in Z_{K}$ by first computing ${\left\langle y\right\rangle }_{i}^{K}={\left\langle x\right\rangle }_{i}^{K}+{\left\langle r\right\rangle }_{i}^{K}$ for i∈{0,1} and then sending these values to each other. Along with the shares of $r\in Z_{K}$, $P_{2}$ also sends the information in Boolean shares telling whether the summation of the shares of *r* wraps so that $P_{0}$ and $P_{1}$ can convert *r* from the ring $Z_{K}$ to the ring $Z_{L}$. Once they reconstruct $y\in Z_{K}$, $P_{0}$ and $P_{1}$ can change the ring of *y* to $Z_{L}$ by adding *K* to one of the shares of *y* if ${\left\langle y\right\rangle }_{0}^{K}+{\left\langle y\right\rangle }_{1}^{K}$ wraps. After conversion, the important detail regarding $y\in Z_{L}$ is to fix the value of it. $P_{0}$ and $P_{1}$ identify if $\left(x+r\right)\in Z_{K}$ wraps using the private compare (PC) method,^22^ and then $P_{0}$, $P_{1}$, or both add *K* to their shares depending on the Boolean share of the outcome of PC. If both add, then this means that there is no addition to the value of $y\in Z_{L}$. At the end, $P_{i}$ subtracts $r_{i}\in Z_{L}$ from $y_{i}\in Z_{L}$ and obtains $x_{i}\in Z_{L}$ for i∈{0,1}.

#### Most significant bit

One of the biggest improvements that we introduce is in the private determination of the MSB of a secret-shared value *x* via MSB. We deftly integrated MOC and PC into MSB so that we could significantly reduce the communication round complexity of it. Given the shares of *x*, MSB first extracts the least significant (n−1)-bits via modK. Then, it converts the ring of this value from $Z_{K}$ to $Z_{L}$ and subtracts it from *x*. This results in either 0 or *K* in $Z_{L}$, and MSB secretly maps it to 0 or 1, respectively, to obtain the MSB of *x*.

#### Comparison

We also provide CMP, which privately compares two secret-shared values, *x* and *y*, and outputs 1 if x>y, and 0 otherwise, in secret-shared form. CMP utilizes MSB to determine the MSB of (x−y) and return the output of MSB as the output of CMP.

#### Multiplexer

We address the private selection via the functionality MUX. It performs the selection of one of two secret-shared values based on the secret-shared selection bit value using the RE of multiplication.^42^ In MUX, the proxies compute the following using their shares (${\left\langle x\right\rangle }_{0}$, ${\left\langle y\right\rangle }_{0}$, ${\left\langle b\right\rangle }_{0}$) and (${\left\langle x\right\rangle }_{1}$, ${\left\langle y\right\rangle }_{1}$, ${\left\langle b\right\rangle }_{1}$):

(Equation 1) $$\begin{array}{l} z=x-b\left(x-y\right)={\left\langle x\right\rangle }_{0}+{\left\langle x\right\rangle }_{1}-{\left\langle b\right\rangle }_{0}\left({\left\langle x\right\rangle }_{0}-{\left\langle y\right\rangle }_{0}\right)-{\left\langle b\right\rangle }_{1}\left({\left\langle x\right\rangle }_{1}-{\left\langle y\right\rangle }_{1}\right)\\ \;-{\left\langle b\right\rangle }_{0}\left({\left\langle x\right\rangle }_{1}-{\left\langle y\right\rangle }_{1}\right)-{\left\langle b\right\rangle }_{1}\left({\left\langle x\right\rangle }_{0}-{\left\langle y\right\rangle }_{0}\right) \end{array}\text{.}$$

Then, they obtain the fresh shares of z=x−b(x−y). As shown in Equation 1, the proxies need to multiply two values owned by different parties in the computation of ${\left\langle b\right\rangle }_{0}\left({\left\langle x\right\rangle }_{1}-{\left\langle y\right\rangle }_{1}\right)$ and ${\left\langle b\right\rangle }_{1}({\left\langle x\right\rangle }_{0}-{\left\langle y\right\rangle }_{0}$. They outsource these multiplications to the helper via the RE. They first prepare six components of the encoding of this function using a set of random values and send four of them to the helper party. The helper party then combines these components in a way that results in a partially decrypted intermediate result. It secret shares this intermediate result to the proxies, and, as the final step, the proxies subtract their share of the intermediate result from the unsent component of the encoding to obtain ${\left\langle b\right\rangle }_{0}\left({\left\langle x\right\rangle }_{1}-{\left\langle y\right\rangle }_{1}\right)$, ${\left\langle b\right\rangle }_{1}({\left\langle x\right\rangle }_{0}-{\left\langle y\right\rangle }_{0}$, and, eventually, the selected value privately. This application of RE demonstrates its potential as a tool for secure MPC protocol design.

#### Matrix product

Since the matrix product is a widely used operation in machine learning algorithms, we provide MP. It essentially uses the same idea of MUL, and we use the same optimization as Wagh et al.^22^ Please refer to that study for further details. Note that we perform dot product operations represented as DP in Figure 2 using MP too.

#### Exponential

Even though the private EXP has been addressed before,^43^^,^^44^ it is inefficient and/or approximates the fractional and negative EXP. In this study, we introduce the exact exponential functionality EXP. It computes the exact exponential of a publicly known base raised to the power of a given secret-shared value, which has been computed by approximation so far in the literature. For this purpose, we have been inspired by the square-and-multiply algorithm and extended the core idea of the square-and-multiply algorithm to cover the exact exponential computation of not only the positive numbers but also the negative numbers as well as their decimal parts in a multi-party scenario. As an overview, the proxies first obtain the MSB of the secret-shared power and use this to select the set containing either the power itself and the contribution of each bit of a positive power or the absolute of the power and the contribution of each bit of a negative power. Then, the proxies determine the value of each bit of the power in a secret-sharing form and use them to select between the previously selected contributions of the bits and a vector of 1s. The last step is to multiply these selected contributions of the bits of the power to the exponential in a binary-tree-like structure. In total, EXP requires two MSB, two MUX, and $log_{2}\left(n\right)$-many MUL. Our exponential can also be extended to address the EXP when the base is also secret shared.

### Security analysis

#### Lemma 1. The protocol MOC securely realizes the functionality $F_{MOC}$ in the $F_{\text{PC}}$ hybrid model

##### Proof

First, we prove the correctness of our protocol by showing $\left({\left\langle x\right\rangle }_{0}^{K}+{\left\langle x\right\rangle }_{1}^{K}\right)\mathrm{mod}\;K=\left({\left\langle x\right\rangle }_{0}+{\left\langle x\right\rangle }_{1}\right)\mathrm{mod}\;L$. In the protocol, y=(x+r)modK and $\text{isWrap}\left(x,r,K\right)=r\overset{?}{>}y$, that is, isWrap(x,r,K)=1 if r>y and 0 otherwise. At the beginning, $P_{0},P_{1}$, and $P_{2}$ call $F_{PC}$ to compute $c=r\overset{?}{>}y$, and $P_{0}$ and $P_{1}$ obtain the Boolean shares $c_{0}$ and $c_{1}$, respectively. Besides, $P_{2}$ also sends the Boolean shares $w_{0}$ and $w_{1}$ of $w=\text{isWrap}\left({\left\langle r\right\rangle }_{0},{\left\langle r\right\rangle }_{1},K\right)$ to $P_{0}$ and $P_{1}$, respectively. If $\text{isWrap}\left({\left\langle y\right\rangle }_{0},{\left\langle y\right\rangle }_{1},K\right)$ is 1, then $P_{0}$ adds *K* to ${\left\langle y\right\rangle }_{0}$ to change the ring of *y* from *K* to *L*. To convert *r* from ring *K* to ring *L*, $P_{0}$ and $P_{1}$ add *K* to their shares of *r* based on their Boolean shares $w_{0}$ and $w_{1}$, respectively. If $w_{0}=1$, then $P_{0}$ adds *K* to its $r_{1}$ and $P_{1}$ does the same with its shares. Later, we need to fix is the summation of *x* and *r*, that is, the value *y*. In the case of x+r≥K, we cannot fix the summation value *y* in ring *L* by simply converting it from ring *K* to ring *L*. This summation should be x+r in ring *L* rather than (x+r) mod *K*. To handle this problem, $P_{0}$ and $P_{1}$ add *K* to their shares of *y* based on their shares $c_{0}$ and $c_{1}$. As a result, we convert the values *y* and *r* to ring *L* and fix the value of *y* if necessary. The final step to obtain $x_{i}$ for party $P_{i}$ is to simply subtract $r_{i}$ from $y_{i}$ where i∈{0,1}.

Next, we prove the security of our protocol. $P_{2}$ involves this protocol in execution of $F_{PC}$. We give the proof $F_{PC}$ above. At the end of the execution of $F_{PC}$, $P_{2}$ learns $u^{\prime }$. However, $u^{\prime }=u⊕\left(x>r\right)$, and $P_{2}$ does not know *u*. Thus, $u^{\prime }$ is uniformly distributed and can be perfectly simulated with randomly generated values. $P_{i}$ where i∈{0,1} sees fresh shares of ${\left\langle r\right\rangle }_{i}^{K}$, ${\left\{{\left\langle r\left[j\right]\right\rangle }_{i}^{V}\right\}}_{j\in \left[n\right]}$, $w_{i}^{B}$, and $u_{i}^{B}$. These values can be perfectly simulated with randomly generated values.

#### Lemma 2. The protocol MSB securely realizes the functionality $F_{MSB}$ in the $F_{\text{MOC}}$ hybrid model

##### Proof

First, we prove the correctness of our protocol. Assume that we have *n*-bit number *u*. $v=u-\left(umod2^{n-1}\right)$ is either 0 or $2^{n-1}$. In our protocol, ${\left\langle z\right\rangle }_{i}$ is the output of $P_{i}$ where i∈{0,1}. We have to prove that $\text{Reconstruct}\left({\left\langle z\right\rangle }_{i}\right)$ is equal to the MSB of *x*. $P_{i}$ where i∈{0,1} computes $d_{i}^{K}=x_{i}\;\mathrm{mod}\;K$, which is a share of *d* over *K*. $P_{i}$ computes $d_{i}$, which is a share of *d* over *L* by invoking MOC. Note that $z=x-\text{Reconstruct}\left({\left\langle d\right\rangle }_{i}\right)$, and all bits of *z* are 0 except the MSB of *z*, which is equal to the MSB of *x*. Now, we have to map *z* to 1 if it is equal to *K* or 0 if it is equal to 0. $P_{0}$ sends the $z_{0}$ and $z_{0}+K$ in random order to $P_{2}$, and $P_{1}$ sends the $z_{1}$ to $P_{2}$. $P_{2}$ reconstructs two different values, divides these values by *K*, creates two additive shares of them, and sends these shares to $P_{0}$ and $P_{1}$. Since $P_{0}$ and $P_{1}$ know the order of the real MSB value, they correctly select the shares of its mapped value.

Second, we prove the security of our protocol. $P_{i}$ where i∈{0,1} sees ${\left\langle d\right\rangle }_{i}$, which is a fresh share of *d*, and ${\left\langle a\left[0\right]\right\rangle }_{i}$ and ${\left\langle a\left[1\right]\right\rangle }_{i}$, one of which is a fresh share of the MSB of *x* and the other is a fresh share of the complement of the MSB of *x*. Thus, the view of $P_{i}$ can be perfectly simulated with randomly generated values.

#### Lemma 3. The protocol CMP securely realizes the functionality $F_{\text{CMP}}$ in the $F_{\text{MSB}}$ hybrid model

##### Proof

First, we prove the correctness of our protocol. Assume that we have *x* and *y*. We first compute z=y−x. If *z* is negative, which corresponds to 1 in the MSB of *z*, then it means that x>y. In this case, CMP outputs 1. If *z* is non-negative, which corresponds to 0 in the MSB of *z*, then it indicates that y≥x. In this case, the output of CMP is 0. Since the output of CMP exactly matches the output of MSB and we have already proved the correctness of MSB, we can conclude that CMP works correctly.

Second, we prove the security of our protocol. Since ${\left\langle z\right\rangle }_{i}={\left\langle y\right\rangle }_{i}-{\left\langle x\right\rangle }_{i}$ is computed locally by $P_{i}$ for i∈{0,1}, it does not reveal any information about *x* and *y*. Afterward, MSB is called on ${\left\langle z\right\rangle }_{i}$ to determine the MSB of *z* in secret-shared form. Considering that the security of MSB is proven, we can conclude that CMP compares two secret-shared values without compromising their privacy.

#### Lemma 4. The protocol MUX securely realizes the functionality $F_{\text{MUX}}$

Proof

We first prove the correctness of our protocol. ${\left\langle z\right\rangle }_{i}$ is the output of $P_{i}$ where i∈{0,1}. We need to prove that $\text{Reconstruct}\left({\left\langle z\right\rangle }_{i}\right)=\left(1-b\right)x+by$.

(Equation 2) $$\begin{array}{l} {\left\langle z\right\rangle }_{0}+{\left\langle z\right\rangle }_{1}={\left\langle x\right\rangle }_{0}-{\left\langle b\right\rangle }_{0}\left({\left\langle x\right\rangle }_{0}-{\left\langle y\right\rangle }_{0}\right)+r_{1}{\left\langle b\right\rangle }_{0}+r_{2}\left({\left\langle x\right\rangle }_{0}-{\left\langle y\right\rangle }_{0}\right)+\\ {\;r}_{2}r_{3}+{\left\langle x\right\rangle }_{1}-{\left\langle b\right\rangle }_{1}\left({\left\langle x\right\rangle }_{1}-{\left\langle y\right\rangle }_{1}\right)+r_{0}\left({\left\langle x\right\rangle }_{1}-{\left\langle y\right\rangle }_{1}\right)+\\ {\;r}_{0}r_{1}+r_{3}{\left\langle b\right\rangle }_{1}-{\left\langle b\right\rangle }_{0}{\left\langle x\right\rangle }_{1}+{\left\langle b\right\rangle }_{0}{\left\langle y\right\rangle }_{1}-{\left\langle b\right\rangle }_{0}r_{1}-r_{0}{\left\langle x\right\rangle }_{1}+\\ {\;r}_{0}{\left\langle y\right\rangle }_{1}-r_{0}r_{1}-{\left\langle x\right\rangle }_{0}{\left\langle b\right\rangle }_{1}-{\left\langle x\right\rangle }_{0}r_{2}+{\left\langle y\right\rangle }_{0}{\left\langle b\right\rangle }_{1}+{\left\langle y\right\rangle }_{0}r_{2}-\\ {\;r}_{3}{\left\langle b\right\rangle }_{1}-r_{3}r_{2}=\left(1-{\left\langle b\right\rangle }_{0}-{\left\langle b\right\rangle }_{1}\right)\left({\left\langle x\right\rangle }_{0}+{\left\langle x\right\rangle }_{1}\right)+\\ \;\left({\left\langle b\right\rangle }_{0}+{\left\langle b\right\rangle }_{1}\right)\left({\left\langle y\right\rangle }_{0}+{\left\langle y\right\rangle }_{1}\right)=\left(1-b\right)x+by \end{array}$$

Next, we prove the security of our protocol. $P_{2}$ gets $M_{2},M_{3},M_{5}$, and $M_{6}$. All these values are uniformly random values because they are generated using uniformly random values $r_{0},r_{1},r_{2},{\text{and}\;r}_{4}$. $P_{2}$ computes $M_{2}M_{5}+M_{3}M_{6}$. The computed value is still uniformly random because it contains uniformly random values $r_{0},r_{1},r_{2},{\text{and}\;r}_{4}$. As a result, any value learned by $P_{2}$ is perfectly simulated. For each i∈{0,1}, $P_{i}$ learns a fresh share of the output. Thus, $P_{i}$ cannot associate the share of the output with the shares of the inputs, and any value learned by $P_{i}$ is perfectly simulatable.

#### Lemma 5. The protocol EXP securely computes the exponential of a publicly known base raised to the power of a secret-shared value

##### Proof

We begin the proof by showing the correctness of the method. Let *x* be the power whose representation in our number format is ⟨x⟩ and *b* be the publicly known base. $P_{0}$ or $P_{1}$ computes $C_{p}=\left\{\ldots ,b^{8},b^{4},b^{2},b,b^{1/2},b^{1/4},b^{1/8},\ldots \right\}$ and $C_{n}=\left\{\ldots ,b^{-8},b^{-4},b^{-2},b^{-1},b^{-1/2},b^{-1/4},b^{-1/8},\ldots \right\}$, and the other generates a corresponding set of 0s for $C_{p}$ and $C_{n}$. These values in $C_{p}$ and $C_{n}$ correspond to $b^{2^{i}}$ and $b^{-1\cdot 2^{i}}$, respectively, for i∈{(n−f),…,2,1,0,−1,−2,…,−f}, assuming that only the corresponding bit value of the power *x* is 1. They choose one of these sets based on the sign of *x* and let *C* be the selected set. Afterward, they must choose between $c_{j}\in C$ and 1 depending on ${\left\langle x\right\rangle }_{j}$ where j∈{0,1,…,n}. For this selection, they use the MSB operation on all cases where each bit of *x* is at the MSB position. This is done by shifting the shares of *x* to the left. Once they have the correct set of contributions, they basically multiply all of those contributions to obtain the result of the exponential. This proves the correctness of EXP.

### Corruption of a proxy

At the beginning, since the adversary corrupting a proxy knows only one share of the power *x*, that is, either $x_{0}$ or $x_{1}$, it cannot infer any information about the other share. The first step of the exponential is to compute the possible contribution of every bit of positive and negative power. This is publicly known. The following step is to select between these contributions depending on the result of MSB(x) by using MUX. Since both MSB and MUX are secure, the adversary can neither infer anything about $x_{1-j}$ nor relate the share of the result it obtains to *x* in general. In the next step, they obtain each bit of *x* in secret-shared form by using MSB and bit shifting on the shares of *x*. Considering the proven security of MSB and the shifting being simply local multiplication of each share by 2, there is no information that the adversary could obtain. Afterward, the proxies select the correct contributions by employing MUX. Since MUX gives the fresh share of what is selected, the adversary cannot associate the inputs with the output. The last step is to multiply these selected contributions via MUL, which is also proven to be secure. Therefore, we can conclude that EXP is secure against a semi-honest adversary corrupting a proxy.

### Corruption of the helper

Since the task of the helper party in the computation of the exponential of a secret-shared power is to either provide multiplication triples or perform the required computation on the masked data, there is nothing that the adversary corrupting the helper party could learn about *x*. Therefore, it is fair to state that EXP is secure against a semi-honest adversary corrupting the helper.

## Supplemental information

Document S1. Table S1 and Notes S1S3

Document S2. Article plus supplemental information
