# Supplementary material for: A privacy-preserving approach for cloud-based protein fold recognition
Source: Patterns (N Y). 2024 Jul 19;5(9):101023. doi: 10.1016/j.patter.2024.101023 (PMC11573750; doi:10.1016/j.patter.2024.101023)
Supplement: Document S1. Table S1 and Notes S1S3 [file mmc1.pdf]

Patterns, Volume *5*

Supplemental information

A privacy-preserving approach for cloud-based protein fold recognition

Ali Burak Ünal, Nico Pfeifer, and Mete Akgün

Supplementary Note 1: Fixed-point Arithmetic

Since machine learning algorithms can require both positive and negative real numbers, we use fixed-point arithmetic to represent numbers. In this number format, the most significant bit is allocated for the sign of the value. 0 represents positive and 1 represents negative values. Besides the most significant bit, a certain number of the least significant bits of this representation are used for the fractional part of the value and the rest expresses the integer part.

To illustrate the number format, let n be the number of bits to represent numbers, f be the number of bits allocated for the fractional part and $\mathbb{S}$ be the set of values that can be represented in this number format, one can convert $x \in \mathbb{R}$ to $\hat{x} \in \mathbb{S}$ as follows:

$$\hat{x} = \begin{cases} \lfloor x * 2^f \rfloor & x \geq 0 \\ 2^n - \lfloor |x * 2^f| \rfloor & x < 0 \end{cases} \tag{1}$$

For example, $x = 3.42$ is represented as $\hat{x} = 112066$, which, if we omit the leading zeros, is 1101101011100001010001 in binary for $f = 20$. The two most significant bits, the 21st and 22nd bits, are used to represent 3 and the remainder represents 0.42.

The choice of f depends on the required precision by the task. If the numbers in the task are mostly small or they need to be as accurate as possible, the precision of the representation of the numbers is crucial. In such a case, a higher value for f is required to allow for more decimal places of the original value, which in return requires sacrifices in the upper limit that can be calculated by the building blocks. However, if the numbers that appear during the process are large, f must be set to lower values in order to allow the integer part to represent larger numbers with fewer decimal places.

Supplementary Note 2: Algorithms

1 **Algorithm** MOC()
  input : P_0 and P_1 hold $\langle x\rangle_0^K$ and $\langle x\rangle_1^K$, respectively
  output: P_0 and P_1 get $\langle x\rangle_0$ and $\langle x\rangle_1$, respectively
2 P_0 and P_1 hold a common random bit u'
3 P_2 picks a random number $r \in \mathbb{Z}_K$ and generates $\langle r\rangle_0^K$, $\langle r\rangle_1^K$, $\{\langle r[j]\rangle_0^V\}_{j\in[n]}$ and $\{\langle r[j]\rangle_1^V\}_{j\in[n]}$.
4 P_2 computes $w =$ isWrap$(\langle r\rangle_0^K, \langle r\rangle_1^K, K)$ and divides w into two boolean shares w_0^B and w_1^B
5 P_2 sends $\langle r\rangle_i^K$, $\{\langle r[j]\rangle_i^V\}_{j\in[n]}$ and w_i^B to P_i, for each $i \in \{0,1\}$
6 For each $i \in \{0,1\}$, P_i executes Steps 7-8
7 $\langle y\rangle_i^K = \langle x\rangle_i^K + \langle r\rangle_i^K$
8 P_i reconstructs y by exchanging shares with P_{1-i}
9 $u_i^B =$ PC$(\{\langle r[j]\rangle_i^V\}_{j\in[n]}, y, u')$
10 P_0 computes $u_i^B = u_i^B \oplus u'$
11 For each $i \in \{0,1\}$, P_i computes $c_i^B = w_i^B \oplus u_i^B$
12 P_0 computes $\langle y\rangle_0 = \langle y\rangle_0^K +$ isWrap$(\langle y\rangle_0^K, \langle y\rangle_1^K, K) \cdot K$
13 P_1 sets $\langle y\rangle_1 = \langle y\rangle_1^K$
14 For each $i \in \{0,1\}$, P_i computes $\langle x\rangle_i = \langle y\rangle_i - (\langle r\rangle_i^K + c_i^B \cdot K)$

Algorithm 1: Modulus Conversion (MOC)

1 **Algorithm** MSB()

input : P_0 and P_1 hold $\langle x\rangle_0$ and $\langle x\rangle_1$, respectively

output: P_0 and P_1 get $\langle z\rangle_0$ and $\langle z\rangle_1$, respectively, where z is equal to 0 if the most significant bit of x is 0 and 1 otherwise.

2 P_0 and P_1 hold a common random bit f and g. P_0 and P_1 additionally hold ℓ common random values $s_j \in \mathbb{Z}_V^*$ for all $j \in [\ell]$, a random permutation π for ℓ elements and ℓ common random values $u_j \in \mathbb{Z}_V^*$.

3 P_2 picks a random number $r \in K$ and generates $\langle r\rangle_0^K$, $\langle r\rangle_1^K$, $\{\langle r[j]\rangle_0^V\}_{j\in[\ell]}$ and $\{\langle r[j]\rangle_1^V\}_{j\in[\ell]}$.

4 P_2 computes $w =$ isWrap$(\langle r\rangle_0^K, \langle r\rangle_1^K, K)$

5 P_2 sends $\langle r\rangle_i^K$ and $\{\langle r[j]\rangle_i^V\}_{j\in[\ell]}$ to P_i, for each $i \in \{0,1\}$

6 For each $i \in \{0,1\}$, P_i executes Steps 7-26

7 $\langle d\rangle_i^K = \langle x\rangle_i \mod K$

8 $\langle y\rangle_i^K = \langle d\rangle_i^K + \langle r\rangle_i^K$

9 $y = \text{Reconst}(\langle y\rangle_i^K)$

10 $\langle y\rangle_i = \langle y\rangle_i^K + i \cdot$ isWrap$(\langle y\rangle_0^K, \langle y\rangle_1^K, K) \cdot K$

11 $\langle a[0]\rangle_i = ifK - \langle x\rangle_i + \langle y\rangle_i - \langle r\rangle_i^K$

12 $\langle a[1]\rangle_i = i(1-f)K - \langle x\rangle_i + \langle y\rangle_i - \langle r\rangle_i^K$

13 Let $t = y + 1 \mod 2^\ell$

14 **for** $j = \ell - 1;\ j > 0;\ j = j - 1$ **do**

15   **if** $g = 0$ **then**

16     $\langle w_j\rangle_i^V = \langle r[j]\rangle_i^V + iy[j] - 2y[j]\langle r[j]\rangle_i^V$

17     $\langle c_j\rangle_i^V = iy[j] - \langle r[j]\rangle_i^V + j + \sum_{k=j+1}^{\ell} \langle w_k\rangle_i^V$

18   **else if** $g = 1$ *AND* $r \neq 2^\ell - 1$ **then**

19     $\langle w_j\rangle_i^V = \langle r[j]\rangle_i^V + it[j] - 2t[j]\langle r[j]\rangle_i^V$

20     $\langle c_j\rangle_i^V = -it[j] + \langle r[j]\rangle_i^V + i + \sum_{k=j+1}^{\ell} \langle w_k\rangle_i^V$;

21   **else**

22   **if** $i \neq 1$ **then**

23       $\langle c_j\rangle_i^V = (1-i)(u_j + 1) - iu_j$

24   **else**

25       $\langle c_j\rangle_i^V = (-1)^j \cdot u_j$

26 Send $\left\{\langle b_j\rangle_i^V\right\}_j = \pi\left(\left\{s_j \langle c_j\rangle_i^V\right\}_j\right)$ and $\langle a\rangle_i$ to P_2

27 For all $j \in [\ell]$, P_2 computes $d_j = \text{Reconst}(\langle d_j\rangle_0^V, \langle d_j\rangle_1^V)$ and sets $g' = 1$ iff $\exists j \in [\ell]$ such that $d_j = 0$.

28 P_2 reconstructs $a[j]$ where $j \in \{0,1\}$ and computes $a[j] = (a[j] - (g' \oplus w)K)/K$

29 P_2 creates two fresh shares of $a[j]$ where $j \in \{0,1\}$ and sends them to P_0 and P_1

30 For each $i \in \{0,1\}$, P_i executes Step 31

31 $\langle z\rangle_i = \langle a[f \oplus g]\rangle_i$

Algorithm 2: Most Significant Bit (MSB)

Supplementary Note 3: Round Complexity and Execution Time Analysis

We give the round complexity of our building blocks and their runtime on different network settings for specific sizes of input data. Even though the purpose of the paper is not to propose a new MPC framework, we compare our building blocks to similar ones in the literature to demonstrate their efficiency. Considering the similarity of the number of parties, the design of these parties, and the secret sharing technique, SecureNN[1] seems the best choice for such a pur-

1 **Algorithm** MUX()

input : P_0 and P_1 hold ($\langle x\rangle_0$, $\langle y\rangle_0$, $\langle b\rangle_0$) and ($\langle x\rangle_1$, $\langle y\rangle_1$, $\langle b\rangle_1$), respectively.

output: P_0 and P_1 get $\langle z\rangle_0$ and $\langle z\rangle_1$, respectively, where $z = x - b(x - y)$.

2 P_0 and P_1 hold four common random values r_i where $i \in \{0, 1, 2, 3\}$

3 P_0 computes $M_1 = \langle x\rangle_0 - \langle b\rangle_0(\langle x\rangle_0 - \langle y\rangle_0) + r_1\langle b\rangle_0 + r_2(\langle x\rangle_0 - \langle y\rangle_0) + r_2r_3$, $M_2 = \langle b\rangle_0 + r_0$, $M_3 = \langle x\rangle_0 - \langle y\rangle_0 + r_3$

4 P_0 sends M_2 and M_3 to P_2

5 P_1 computes $M_4 = \langle x\rangle_1 - \langle b\rangle_1(\langle x\rangle_1 - \langle y\rangle_1) + r_0(\langle x\rangle_1 - \langle y\rangle_1) + r_0r_1 + r_3\langle b\rangle_1$, $M_5 = (\langle x\rangle_1 - \langle y\rangle_1) + r_1$, $M_6 = \langle b\rangle_1 + r_2$

6 P_1 sends M_5 and M_6 to P_2

7 P_2 computes $M_2M_5 + M_3M_6 = z$

8 P_2 divides z into two shares ($\langle z\rangle_0 + \langle z\rangle_1$) and sends $\langle z\rangle_0$ and $\langle z\rangle_1$ to P_0 and P_1, respectively

9 P_0 computes $\langle z\rangle_0 = M_1 - \langle z\rangle_0$

10 P_1 computes $\langle z\rangle_1 = M_4 - \langle z\rangle_1$

Algorithm 3: Multiplexer (MUX)

1 **Algorithm** EXP()

input : P_0 and P_1 hold $\langle x\rangle_0$ and $\langle x\rangle_1$, respectively, and publicly known base b

output: P_0 and P_1 get $\langle z\rangle_0$ and $\langle z\rangle_1$, respectively

2 For $i \in \{0, 1\}$, P_i executes Steps 3-15 with the help of P_2

3 $\langle s\rangle_i$ = MSB($\langle x\rangle_i$)

4 $\langle |x|\rangle_i = 0 - \langle x\rangle_i$

5 **for** $j = n;\ j > 0;\ j = j - 1$ **do**

6 $\quad\langle cP[n-j]\rangle_i = i * b^{2^{j-f}}$

7 $\quad\langle cN[n-j]\rangle_i = i * (1/b^{2^{j-f}})$

8 $(\langle \hat{x}\rangle_i, \langle cONE\rangle_i)$ = MUX$((\langle x\rangle_i, \langle cP\rangle_i), (\langle |x|\rangle_i, \langle cN\rangle_i), \langle s\rangle_i)$

9 **for** $j = 0;\ j < n;\ j = j + 1$ **do**

10 $\quad\langle \hat{x}^{n-j}\rangle_i = (\langle \hat{x}\rangle_i << j)$

11 $\langle M\rangle_i$ = MSB$((\langle \hat{x}^{64}\rangle_i, \langle \hat{x}^{63}\rangle_i, \ldots, \langle \hat{x}^{1}\rangle_i))$

12 $\langle cACT\rangle_i$ = MUX$(\langle cONE\rangle_i, \langle cZERO\rangle_i, \langle M\rangle_i)$

13 $\langle z\rangle_i = \langle cACT\rangle_i$

14 **for** $j = 0;\ j < log_2(n);\ j = j + 1$ **do**

15 $\quad\langle z\rangle_i$ = MUL$(\langle z[0 : len(z)/2]\rangle_i, \langle z[len(z)/2 : len(z)]\rangle_i)$

Algorithm 4: Exponential computation (EXP)

pose. In our MSB, for instance, we need 4 rounds whereas it requires 5 rounds in SecureNN. Besides, MSB of SecureNN takes the input from the ring $L - 1$ and outputs in the ring L where $L = 2^{64}$. Such inconsistency between the input and the output increases the round complexities of some operations relying on MSB in SecureNN. For example, we can compare the DReLU in SecureNN with our CMP method. We did not include the implementation of the DReLU as it is not necessary for RKN inference. However, DReLU can be considered as the inverse or complement of the CMP process. In DReLU(x), the output is 0 if x is less than 0, and 1 otherwise. On the other hand, CMP(x, y) outputs 1 if $(y - x)$ is less than 0, and 0 otherwise. The calculation of $(y - x)$ is done locally without the need for any communication between the computing parties. The complement of bits in arithmetic secret sharing can be achieved through the subtraction operation, which can be performed locally without requiring communication between the parties. It is evident that these two operations are similar in terms of communication cost and require the same underlying functions. In SecureNN, the DReLU function invokes ShareConvert, which

converts shares from the ring L to the ring $L-1$ in 4 communication rounds. This conversion is necessary because the subsequent function MSB expects inputs in the ring $L-1$, while the inputs to DReLU are in the ring L. As a result, DReLU in SecureNN requires 8 communication rounds, which is 1 less than the summation of the round complexities of $ShareConvert$ and MSB thanks to an optimization that they did while combining. On the other hand, our CMP function only invokes our MSB function, which takes inputs and gives outputs in the ring L without requiring share conversion. Therefore, our CMP only requires 4 communication rounds. **(Table S1)** summarizes the communication round complexities of the utilized building blocks.

It is important to note that even though we have some methods with similar names to the methods in SecureNN, they, in fact, have completely different functionalities. For instance, our MOC method involves converting shares from the 2^{63} ring to the 2^{64} ring. In SecureNN, there is no equivalent method to MOC. The closest method in SecureNN is ShareConvert, which converts shares from the 2^{64} ring to the $(2^{64})-1$ ring. Although both methods require 4 rounds of communication, they differ significantly in terms of input and methodology.

Protocol	RC	LAN Time (ms)	WAN Time (ms)	
			20 ms delay	58 ms delay
MUL	2	7.9	25.6	64.1
MUX	2	6.53	48.2	127
MOC	4	307	377	537
MSB	4	65.6	133	264
CMP	4	68.1	145	264
EXP	12 + 2 $\log n$	536	839	1380

Table S1: Round complexities (RC) of our building blocks where n is the number of bits to represent values. In addition to the round complexity, we also give the runtime of our building blocks on different network settings for input vectors of size 10^5.

References

1. Wagh, S., Gupta, D., and Chandran, N. (2019). SecureNN: 3-Party Secure Computation for Neural Network Training. Proc. Priv. Enhancing Technol. *2019*, 26–49.
