# Supplementary material for: A privacy-preserving approach for cloud-based protein fold recognition
Source: Patterns (N Y). 2024 Jul 19;5(9):101023. doi: 10.1016/j.patter.2024.101023 (PMC11573750; doi:10.1016/j.patter.2024.101023)
Supplement: Document S2. Article plus supplemental information [file mmc2.pdf]

Patterns

Article

A privacy-preserving approach for cloud-based protein fold recognition

Highlights

- We propose private machine learning as a service for protein fold recognition
- We combine recurrent kernel networks and multi-party computation
- Our approach computes the same result as plaintext RKN without compromising privacy
- We show its linear scalability to the parameters of recurrent kernel networks

Authors

Ali Burak Ünal, Nico Pfeifer, Mete Akgün

Correspondence

ali-burak.uenal@uni-tuebingen.de (A.B.Ü.), nico.pfeifer@uni-tuebingen.de (N.P.), mete.akguen@uni-tuebingen.de (M.A.)

In brief

This work proposes the first privacy-preserving machine-learning-as-a-service approach for protein fold recognition tasks. It utilizes multi-party computation to perform inference on the query sequence via pre-trained recurrent neural networks. The authors design and implement several efficient multi-party computation building blocks to address the required operations in recurrent kernel networks. They demonstrate its correctness on the Structural Classification of Proteins dataset and the scalability of the solution to various parameters on a synthetic dataset.

Ünal et al., 2024, Patterns *5*, 101023
September 13, 2024 © 2024 The Author(s). Published by Elsevier Inc.
https://doi.org/10.1016/j.patter.2024.101023

CellPress

Patterns

CellPress
OPEN ACCESS

Article

A privacy-preserving approach for cloud-based protein fold recognition

Ali Burak Ünal,[1,3,4,*] Nico Pfeifer,[2,3,*] and Mete Akgün[1,3,*]
[1]Medical Data Privacy and Privacy Preserving Machine Learning (MDPPML), Department of Computer Science, University of Tübingen, 72076 Tübingen, Germany
[2]Methods in Medical Informatics, Department of Computer Science, University of Tübingen, 72076 Tübingen, Germany
[3]Institute for Bioinformatics and Medical Informatics (IBMI), Department of Computer Science, University of Tübingen, 72076 Tübingen, Germany
[4]Lead contact
*Correspondence: ali-burak.uenal@uni-tuebingen.de (A.B.Ü.), nico.pfeifer@uni-tuebingen.de (N.P.), mete.akguen@uni-tuebingen.de (M.A.)
https://doi.org/10.1016/j.patter.2024.101023

THE BIGGER PICTURE In the era of cloud-based machine learning, privacy concerns, especially in medicine, are critical. Protecting the privacy of medical data is essential for maintaining patient trust and complying with regulations. Recognizing protein folds is vital for understanding diseases and developing treatments, but it currently lacks a privacy-preserving solution. We present an approach that secures this process, allowing the use of advanced models without compromising data or model privacy. By maintaining high performance while ensuring privacy, our scalable and efficient solution demonstrates the practicality of secure cloud-based machine learning in healthcare. This work highlights the urgent need for privacy-conscious cloud-based machine learning and aims to inspire further advancements, emphasizing the importance of data privacy in medical applications.

SUMMARY

The complexity and cost of training machine learning models have made cloud-based machine learning as a service (MLaaS) attractive for businesses and researchers. MLaaS eliminates the need for in-house expertise by providing pre-built models and infrastructure. However, it raises data privacy and model security concerns, especially in medical fields like protein fold recognition. We propose a secure three-party computation-based MLaaS solution for privacy-preserving protein fold recognition, protecting both sequence and model privacy. Our efficient private building blocks enable complex operations privately, including addition, multiplication, multiplexer with a different methodology, most-significant bit, modulus conversion, and exact exponential operations. We demonstrate our privacy-preserving recurrent kernel network (RKN) solution, showing that it matches the performance of non-private models. Our scalability analysis indicates linear scalability with RKN parameters, making it viable for real-world deployment. This solution holds promise for converting other medical domain machine learning algorithms to privacy-preserving MLaaS using our building blocks.

INTRODUCTION

Machine learning as a service (MLaaS) has become so popular recently due to its efficiency and practicality in various domains. With the increased complexity and cost of training machine learning algorithms, it has become challenging for businesses and researchers to develop and deploy these models in-house, as such an action would require considerable expertise and computational power. Cloud-based MLaaS solutions provide access to pre-trained models, avoiding the need for expensive hardware and software investments and reducing the time and resources needed to develop a model from scratch. Thanks to its efficiency and practicality, MLaaS has been successfully applied to various domains,[1–3] including the medical domain.

One specific problem in the medical domain is the protein fold recognition task. The structure of a protein is one of the factors determining its functionality.[4,5] The shape of a protein, for instance, affects its ability to bind to other proteins.[6,7] One of the steps toward modeling the structure of a protein is to determine the folds of a protein by comparing the given protein sequence to the sequences of proteins with known structures.[8] By this approach, one can predict the structure of a protein

This is an open access article under the CC BY license (http://creativecommons.org/licenses/by/4.0/).

and assess its functionality to some extent. As an illustrative example of why such information is important, consider a patient with mutations in several of their genes. Determining whether these mutations affect the structure of the proteins that are synthesized based on these genes can help physicians select the correct treatment for the patient, leading to literally a life-or-death decision. In the literature, there are several different approaches proposed for protein fold recognition such as DeepSVM-Fold,[9] DeepMSA,[10] AlphaFold,[11,12] ESMFold,[13] and recurrent kernel networks (RKNs).[14] Among these approaches, even though AlphaFold and ESMFold are MLaaS solutions for protein fold recognition, they do not utilize any privacy-enhancing technique and, as a natural outcome, protect the sensitive data in the query sequences. To the best of our knowledge, the submitted query sequence to AlphaFold and ESMFold can be accessible in plaintext by the server. This access to the protein sequences poses a significant challenge. The sequences contain sensitive information about the individual from whom they were derived, such as genetic predispositions to certain diseases, and this information about the corresponding individual can be compromised by the server owner. In addition to the input sequence's privacy, the model's privacy can be an issue if the model is proprietary and the owner of the model does not have enough computational power to provide MLaaS. To allow others to benefit from the model, the owner has to outsource the model to a third party. However, outsourcing the model in plaintext risks the intellectual property of the model, and the third party could use the model in additional scenarios without the knowledge of the developer of the model. In summary, even though there exist some MLaaS protein fold recognition approaches, to the best of our knowledge, there exists no privacy-preserving protein fold recognition approach proposed in the literature.

To address the need to protect the privacy of the protein sequences and the model, a natural path is to integrate a privacy-enhancing technique into the process. Various privacy-enhancing techniques have been proposed in the literature to protect sensitive data during such operations. One of these techniques is differential privacy (DP). It introduces noise to a phase or several phases of machine learning training and/or testing to protect the privacy of the data and the model.[15,16] However, DP can significantly reduce the accuracy of a model since its main mechanism to provide privacy is to add noise to the data/model parameters. Another technique is homomorphic encryption (HE), where all the computations are performed on encrypted data.[17–20] The computations on the homomorphically encrypted data do not reveal any information about the underlying data thanks to their encrypted nature. However, the limited operations offered by HE and its computational expensiveness make it impractical for real-world applications. Secure multiparty computation (MPC), however, addresses the missing points of HE and the fundamental privacy requirements. The data and the model parameters are shared among several parties in such a way that none of the parties can learn about the data and/or the model parameters on their own. Then, these parties perform the desired computation privately. To address various machine learning algorithms, there are several MPC frameworks in the literature,[21–27] some of which also utilize HE.[28,29] Their focus is, however, to address mostly convolutional neural network (CNN) models in a privacy-preserving way, and the building blocks of these MPC frameworks are customized to perform CNN operations efficiently.

Compared to the large architecture and complexity of AlphaFold and ESMFold, RKNs[14] have a more privacy-friendly deep learning architecture. Chen et al.[14] gave a kernel perspective of recurrent neural networks (RNNs) by showing that the computation of the specific construction of RNNs, which they call RKNs, mimics the substring kernel allowing mismatches and the local alignment kernel, which are widely used on sequence data.[30–32] In RKNs, small motifs called anchor points are used as templates to measure similarities among sequences. By traversing every character of the sequence, the overall search for a mapping of anchor points is performed, and the final mapping of the sequence is computed by multiplying the initial mapping and the inverse square root of the gram matrix of the anchor points. Then, the classifier layer gives the prediction score of the sequence. Thanks to the combination of a well-designed kernel formulation and parameter optimization through backpropagation, RKNs outperform the traditional substring kernel and the local alignment kernel, as well as long short-term memories (LSTMs).[33]

Considering the merits of RKNs and well-combined privacy and computational efficiency features of MPC, in this paper, we address the necessity of private and secure MLaaS for protein fold recognition by proposing privacy-preserving RKN as a service using MPC. In our solution, an overview of which is given in Figure 1, we perform protein fold recognition on a given sequence without sacrificing the privacy of the sequence or the model. More specifically, both the input sequence and model parameters are secret shared to computing parties so that neither the model owner nor the sequence owner has to sacrifice their confidential information for the sake of the inference. The realization of these operations accurately via existing MPC frameworks, however, is a challenging task, if not impossible. Many MPC frameworks in the literature are designed for CNNs, and it is difficult to adapt them to new problems due to a lack of documentation and code flexibility. While we benefit from existing basic MPC operations such as addition and multiplication to perform the privacy-preserving RKN as a service, we design and implement several highly efficient MPC building blocks to perform the classification of the given protein sequence on the outsourced pre-trained RKN model without compromising the privacy of the sequence data or the model parameters. We call the resulting MPC framework CECILIA. As a summary, our contributions can be listed as follows.

- Privacy-preserving RKN as a service: we propose the privacy-preserving MLaaS protein fold recognition approach based on RKNs. The performance of RKNs, thanks to their well-established kernel method basis and backpropagation—allowing deep neural network yet privacy-friendly architecture—made RKN the best choice for this task.
- Efficient MPC building blocks: considering the lack of comprehensive documentation, flexibility, and user-friendly interfaces of the existing MPC frameworks, we designed and implemented highly efficient MPC primitives, resulting in an MPC framework, CECILIA, to address these limitations. These primitives include the conversion of shares from the 2^{n-1} ring to the 2^n ring, known as modulus conversion (MOC), the computation of the most significant bit (MSB) of

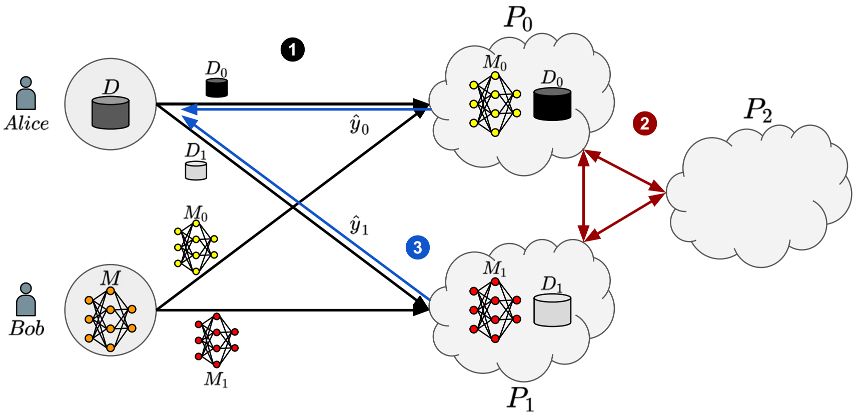

Figure 1. The overview of our privacy-preserving RKN as a service via MPC
(1) At first, the data owner, i.e., Alice, secret shares her data and sends the shares to the proxies. Similarly, the model owner, i.e., Bob, does the same with the parameters of the model. (2) Then, by using the outsourced model and the data, the proxies, P_0 and P_1, perform the operations required for the inference of the data on the model with the help of P_2, which is the helper. (3) Finally, the proxies send the shares of the prediction of the given data to the data owner.

secret-shared values, the randomized encoding (RE)-based secret-shared multiplexer (MUX) to select, and the secret-shared accurate exponentiation (EXP).

RESULTS

Overview of RKN as a service

Setup

The setup starts with outsourcing. In the outsourcing of the model parameters, which are the anchor point matrix, the linear classifier weights, and the inverse square root of the gram matrices of the anchor points, the model owner secret shares them and sends these shares to the proxies, which are two of three computing parties interacting with the users, such that each proxy has a single share of each parameter. To outsource the test samples, the data owner proceeds similarly after using one-hot encoding to convert a sequence into a vector of numbers. It divides this vector into two shares and sends them to the proxies. Besides outsourcing, the proxies agree on a common seed to generate common randoms.

Private inference

After the setup, we use the building blocks for private inference on a pre-trained RKN as MLaaS, whose internal computations are given in Figure 2. Let $t \in \{1, \ldots, |x|\}$ be the index of the characters in the sequence x. First, the proxies compute $b_j[t]$, which is the similarity of the one-hot-encoded t-th character of the sequence to the j-th character of each anchor point for $j \in \{1, \ldots, k\}$. As shown in the gray boxes in Figure 2A, this calculation involves the dot product of two secret-shared vectors, the subtraction of a plaintext scalar value from a secret-shared value, the multiplication of a plaintext scalar value by a secret-shared value, and the exponential of a known base raised to the power of a secret-shared value. Once the similarity computation is complete, the proxies proceed with the private element-wise product between $b_j[t]$ and $c_{j-1}[t-1]$, which is the initial mapping of the sequence up to the $(t-1)$-th character based on the anchor points of length $(j-1)$. Then, the proxies add the result of the element-wise product with the downgraded $c_j[t-1]$ with a plaintext scalar value λ. At the end of this computation, the proxies obtain the secret-shared initial mapping of the sequence $c_j[t]$ up to the t-th character to q-dimensional space based on each anchor point of length $j \in \{1,\ldots,k\}$.

After computing the full initial mapping of the sequence, that is, $c_k[|x|]$, the proxies multiply the inverse square root of the gram matrices $K_{Z_kZ_k}^{-1/2}$ by the corresponding initial mapping vectors of the sequence. Afterward, the proxies compute the private dot product of two secret-shared vectors, which are the weights of the classifier and the mapping of the sequence. In the end, they obtain the secret-shared prediction of the given sequence. These shares can then be sent back to the owner of the data, enabling the reconstruction of the prediction.

Dataset

To perform the protein fold recognition on RKN as a service, we utilized Structural Classification of Proteins (SCOP) v.1.67,[34] which was also used by Chen et al.[14] It contains 85-fold recognition tasks with positive or negative labels and protein sequences of varying lengths. The comparison of the predictions of our privacy-preserving RKN as a service and plaintext RKN on SCOP demonstrates the correctness of our approach. We also analyze the scalability of it to various parameters of RKN. For this purpose, we use a synthetic dataset.

Experimental setup

We conducted the experiments on a dedicated server with an Intel Xeon Gold 6140 CPU running at 2.30 GHz, equipped with 256 GB of memory, and running Ubuntu. We ran our experiments on local area network (LAN) and wide area network (WAN) settings. To simulate the WAN setting, we set the average round-trip time of the local host to 20 ms. We represent numbers with 64 bits whose least significant 20 bits are used for the fractional part.

Experimental evaluation

Correctness analysis

We selected tasks of SCOP and trained an RKN on them using the same parameter setting as Chen et al.,[14] that is, $q = 128$ and $k = 10$. Then, we outsource the parameters of the model to the proxies, which are the anchor points, biases, inverse square root of the anchor points, and linear classifier. All computing parties are connected via LAN. To perform the classification of the protein sequences in the test set of the selected task, we outsource those sequences to the proxies as well. Once the model and the test samples are outsourced, the proxies perform the sequence of required operations and obtain the results in secret-shared form. Then, they return the shares of these results, and we reconstruct them as plaintext. When we

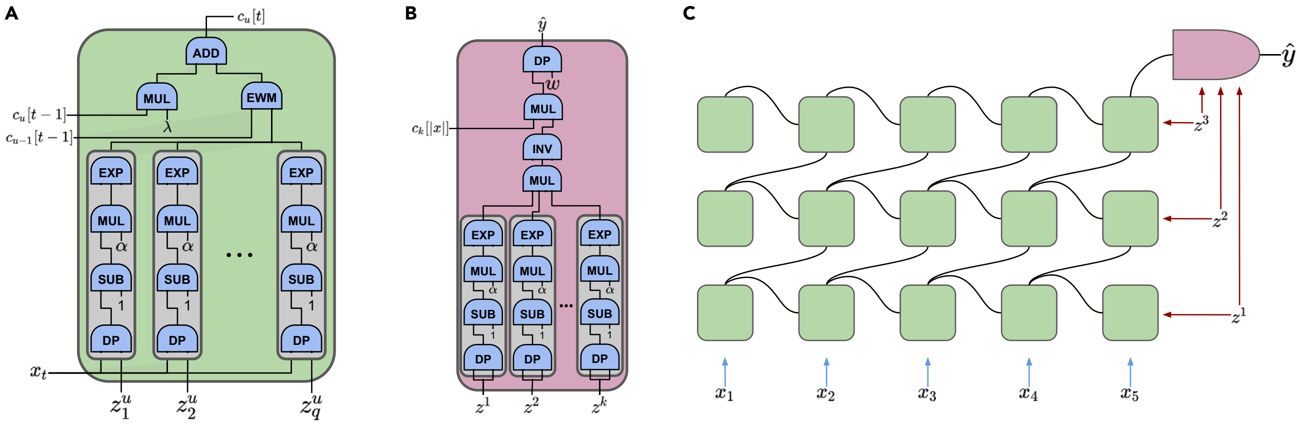

Figure 2. The architecture of RKN and internal computations of its layers

(A and B) The arithmetic circuits of (A) a single neuron of RKN at position t and k-mer level u and (B) the linear classifier layer of RKN after the last position of the input sequence x are depicted. z_i^j represents the i-th character of the j-th anchor point, and $c_i[j]$ represents the initial mapping of the sequence up to its j-th character into a q-dimensional vector based on anchor points of length i where $i \in \{1,\ldots,k\}$, $j \in \{1,\ldots,s\}$, and q is the number of anchor points.

(C) For $k = 3$ and $|x| = 5$, RKN is shown where the green nodes are the single neurons and the pink one is the linear classifier.

compared the predictions of our privacy-preserving RKN as a service with the predictions of the plaintext RKN model, the largest absolute difference between the corresponding predictions is less than 2×10^{-5}, which is an expected difference with fixed-point arithmetic. Such close predictions suggest that our MPC-based RKN as a service can yield the correct results without compromising the privacy of the protein sequence or the model parameters. To give a better perspective on how an inference in real-life deployment would look considering that these sequences are real sequences, we analyze the runtime of performing inference on these sequences. On average, an inference of a query sequence via RKN as a service takes 3.9 s, while the corresponding plaintext inference takes around 3.6×10^{-5} s. Considering the required communication between computing parties, the complexity of operations, and the cumbersome nature of getting the required permissions to use the data in plaintext, if possible at all, it is fair to state that our runtime is acceptable. In the case of extending the capability of this service to respond to multiple requests at the same time via parallelization, the total amount of time required to perform inference on a set of query sequences can be further reduced.

Execution time analysis

We examined the effects of the parameters of the RKN, namely the number of anchor points, the length of the k-mers, and the sequence length, on the execution time of our RKN as a service on both LAN and WAN. To do this, we curated datasets of synthetic protein sequences, focusing on the runtime of the classification rather than its correctness. For the analysis of the number of anchor points and the length of the k-mers, we used a dataset of fixed-length sequences, specifically 128 amino acids for each sequence. To observe the impact of sequence length on execution time, we created a dataset with varying sequence lengths. In our analyses, we varied the parameter of interest while keeping the others fixed for better observation. When analyzing the impact of the number of anchor points on execution time using fixed-length protein sequences, we set the length of k-mers to 8. Similarly, we fixed the number of anchor points to 8 to analyze the impact of the length of k-mers on execution time. To observe the impact of the sequence length, we fixed both the number of anchor points and the length of k-mers to 8. We repeated each experiment 5 times and report their results to have a robust and fair evaluation. Figure 3 summarizes the results of these experiments and illustrates the linear trend in the execution time of the privacy-preserving RKN as a service for different parameters on both LAN and WAN settings.

DISCUSSION

In this study, we introduce the privacy-preserving protein fold recognition in MLaaS by proposing privacy-preserving RKN as a service using MPC. We address the privacy issue in protein fold recognition, which has been overlooked in the literature so far. None of the deployed MLaaS protein fold recognition algorithms have considered the security of the query protein sequence. Thanks to our MPC-based solution, MLaaS protein fold recognition without revealing the query sequence is possible for all sorts of entities and individuals, for some of whom it could have not been possible due to privacy concerns and regulations. A hospital, for instance, could not benefit from a non-private MLaaS protein fold recognition algorithm due to data protection and data security regulations. The hospital is not allowed to send the data of patients outside in plaintext, considering that such an action would compromise the sensitive information of patients to third parties. The structure would be important in scenarios where it is expected that a change in the protein sequence of the patient will lead to a change in the protein structure, but this means that the information can be very sensitive if the change in the protein sequence leads to a change in the structure, which leads to a loss or gain of function of the protein and, potentially, to a disease.[34–37] Therefore, unless the query protein sequence is kept private during the whole operation, such protein fold recognition services have no use for entities and individuals who have privacy concerns. In our solution, we preserve the privacy of the query sequence during the whole

CellPress
OPEN ACCESS

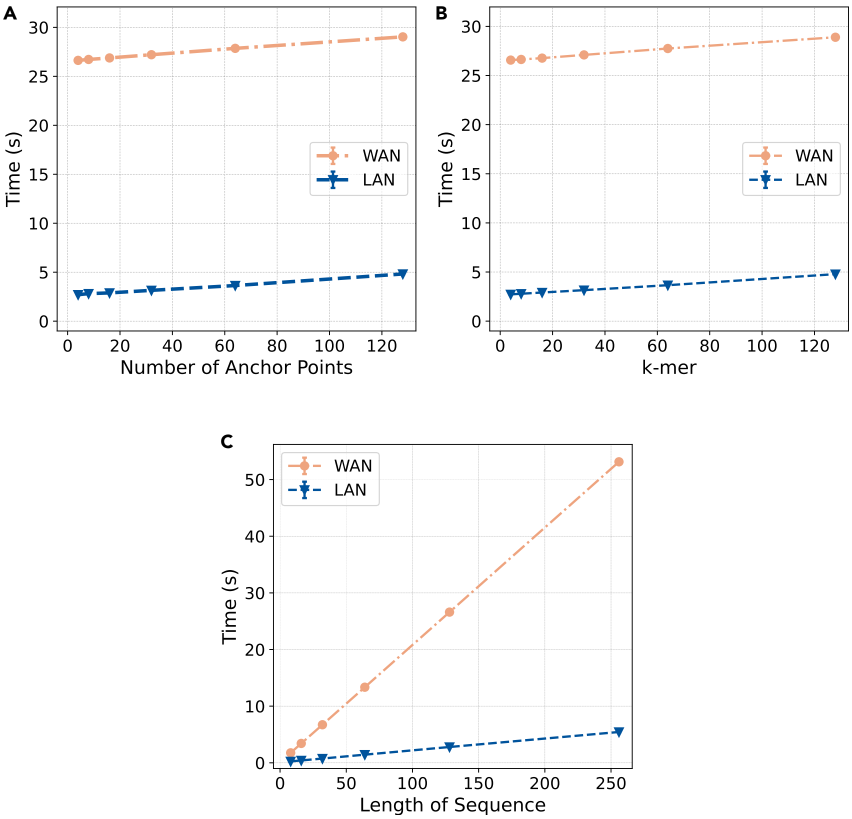

Figure 3. The results of the execution time analysis of our RKN as a service

The results of the execution time analysis of our RKN as a service on both WAN and LAN settings for varying (A) numbers of anchor points for a fixed k-mer length and sequence length, (B) lengths of k-mers for a fixed number of anchor points and sequence length, and (C) lengths of sequences for a fixed number of anchor points and k-mer length.

inference process, and none of the sensitive information is revealed to the computing parties. This will allow the utilization of our MLaaS protein fold recognition algorithm to be used by anyone with a protein sequence.

RKN as a service utilizes MPC to ensure that the sensitive information of the query protein sequence is not compromised. It also addresses the protection of the model parameter privacy, which is especially important when the model is proprietary and outsourced to third parties to provide MLaaS protein fold recognition. In RKN as a service, both the query protein sequence and the model parameters are secret shared to two proxies, allowing them to hold only a single share that does not reveal anything about the original value. They then use randomization to mask the data and perform the required operations with the help of the third party. In the end, they obtain the result in secret-shared form, meaning that they cannot learn the prediction of the RKN model for the given protein sequence. Only the owner can recover the prediction in plaintext after receiving these shares back from the computing parties.

The choice of MPC to provide security and privacy allows us to perform the protein fold recognition task efficiently compared to HE and accurately compared to DP. While providing complete security, MPC is more efficient than HE, allowing us to realize the required operations for RKN as a service in a feasible time frame, as stated in the experimental evaluation section. This makes our MPC-based RKN-as-a-service solution favorable over possible HE-based solutions when the efficiency of the solution is a key criterion. Compared to DP, our MPC-based solution provides more accurate results thanks to its exact computation. Due to the noise addition in DP, the result differs from the result that one would obtain in plaintext protein fold recognition using the same model and the query sequence. Moreover, adding a sufficient amount of noise to the query sequence is not possible when one-hot encoding is used without destroying the one-hot encoding completely. Since the values of the encoding are known to be either 0 or 1, a small amount of noise would not be able to hide these values. A large amount noise, on the other hand, would completely destroy the one-hot encoding, leading to a significant performance loss of the model. This issue can be resolved by making the noisy model publicly available such that the users can use this model locally. However, this contradicts the idea of MLaaS solutions. In summary, when the accuracy of the deployed model is prioritized, our MPC-based solution stands out among possible DP-based solutions.

Our analyses demonstrate the efficiency and accuracy of RKN as a service. In our experiments with SCOP, we have shown that the privacy-preserving RKN as a service is capable of making the same predictions as its plaintext, non-private counterpart. This proves an improvement in the existing methodology, as we do not sacrifice the accuracy of the protein fold recognition model for the sake of privacy, as would be the case for approaches based on $\epsilon - $DP. Instead, we maintain the model's performance while ensuring the privacy of both the input sequence and the model parameters. Moreover, our experiments with synthetic data have shown that the privacy-preserving RKN as a service scales linearly with the number of anchor points, the length of k-mers, and the length of the input sequence, as shown in Figure 3. The privacy-preserving RKN as a service requires a reasonable amount of time to perform inference on the input sequence, suggesting that it can be deployed in real-life scenarios to protect sensitive information in the input sequence and the model. A detailed analysis of communication round complexity of the MPC building blocks can be found in Table S1.

Limitations of the study

While we enable the privacy-preserving inference on a pre-trained RKN model that is outsourced to the proxies, we do not provide the users with the option of fine-tuning this model for their specific tasks. To achieve a meaningful improvement in the model performance via fine-tuning, the anchor points

acting as a template to measure the similarities between sequences need to be fine-tuned, leading to training the network again. However, due to the complexity and uniqueness of RKN operations, this is infeasible using the existing MPC protocols. For instance, one has to be able to compute the inverse square root of the kernel matrix of anchor points, which has not been addressed in the literature. Such a limitation of our solution could be disadvantageous when a user requires a slightly specialized model for their needs and using the existing model could lead to wrong results. One *ad hoc* solution to this issue is to retrain or fine-tune the model using a dataset containing sequences similar to the ones that the user has. Afterward, this newly trained/fine-tuned model can be outsourced to the computing servers, and the user and other users with similar sequences can utilize this model to perform protein fold recognition. Even though such an *ad hoc* solution may solve the problem in some cases, there are cases where such a dataset may not be available for the model owner to retrain/fine-tune the existing model. Therefore, an efficient solution to fine-tuning the existing model for individual users remains an open research problem. Furthermore, we consider only the semi-honest adversary, in other words, the honest-but-curious threat model. Extending our current solution to the malicious adversary is a relatively challenging task. One of the main reasons is the underlying 2-out-of-2 additive secret sharing. In this secret-sharing scheme, a secret value is secret shared using two unique values, making designing maliciously secure MPC protocols highly challenging. First and most importantly, this system cannot provide security in the case of two malicious proxies, that is, the computing parties with the secret shares. They can easily reconstruct the secret value when they are both malicious. In the case of having a malicious proxy and a malicious helper, they can also retrieve the secret value during the computation. For instance, the helper provides random values, which are called multiplication triples, to the proxies during the multiplication operation. In the process, the proxies reconstruct the secret value masked using these random values. Knowing these random values and the masked secret value allows the malicious adversary corrupting the helper and a proxy to obtain the secret value. Therefore, the current system cannot handle a malicious majority. This means that there could be at most a single malicious adversary in the system. Even though a single malicious adversary cannot retrieve the secret value, it can still lead to an incorrect result. A general approach to address this issue in the presence of a malicious adversary in the honest-majority setting in the 2-out-of-2 additive secret-sharing scheme is to perform extra side operations to verify the correctness of the result. This approach is called verifiable computing, consisting of different techniques such as cut and choose.[38] However, this would lead to too many extra computations and, naturally, significant overhead in the runtime. A possible future research direction would be to perform the privacy-preserving RKN as a service in the presence of a malicious adversary without introducing too much extra computation into the current solution.

Overall, our privacy-preserving RKN as a service allows entities and individuals to use an MLaaS protein fold recognition algorithm without compromising the sensitive information of the input sequence in the presence of a semi-honest adversary. Such privacy protection is not just for the users of this model but also for the owner of the model. The owner of the RKN model does not have to reveal the model to deploy the model as an MLaaS either.

EXPERIMENTAL PROCEDURES

Resource availability

Lead contact

Further information and requests for resources should be directed to the lead contact, Ali Burak Ünal (ali-burak.unal@uni-tuebingen.de).

Materials availability

No new biological materials were generated by this study.

Data and code availability

This study uses previously published datasets. We refer to the reader to Murzin et al.[39] to obtain the SCOP dataset and Chen et al.[14] for data preparation steps. Considering that the purpose of the synthetic dataset is to analyze the runtime, not the correctness, we generated the synthetic dataset randomly at runtime. The source code of our privacy-preserving RKN-as-a-service solution is available at Github (https://github.com/mdppml/RKN-as-a-Service) and has been archived at Zenodo.[40]

MPC

MPC is one of the privacy-enhancing techniques based on cryptography where the owner of a secret input secret shares it to two or more computing parties. These computing parties collaborate to collectively compute a function without revealing any participant's complete input. The secret-sharing mechanism ensures that sensitive information remains private, allowing for joint computations while preserving the confidentiality of individual inputs. In 2-out-of-2 additive secret sharing, for instance, the participants divide their inputs into two values such that the individual shares do not reveal anything about the other share and their summation over a specific ring gives the secret input. MPC is especially useful in scenarios where multiple parties need to collaborate on data analysis, and the computation is outsourced to external entities while maintaining the privacy of the original inputs.

Notations

We use 2-out-of-2 additive secret sharing over three different rings, $\mathbb{Z}_L$, $\mathbb{Z}_K$, and $\mathbb{Z}_V$, where $L = 2^n, K = 2^{n-1}, V = 67$, and $n = 64$. We denote two shares of x over $\mathbb{Z}_L$, $\mathbb{Z}_K$, and $\mathbb{Z}_V$ by $(\langle x\rangle_0, \langle x\rangle_1)$, $(\langle x\rangle_0^K, \langle x\rangle_1^K)$, and $(\langle x\rangle_0^V, \langle x\rangle_1^V)$, respectively. If a value x is shared over the ring $\mathbb{Z}_V$, then every bit of x is additively shared in $\mathbb{Z}_V$. This means x is shared as a vector of n shares, where each share takes a value between 0 and $(V-1)$. We also use Boolean sharing of a single bit denoted by $(\langle x\rangle_0^B, \langle x\rangle_1^B)$.

MPC building blocks

The building blocks of our solutions are based on three computing parties and 2-out-of-2 additive secret sharing. In the resulting MPC framework, which we call CECILIA, two of these parties, P_0 and P_1, are called proxies, and the external entities such as the model owner and the data sources interact with them. The third one, P_2, is the helper party, helping the proxies compute the desired function without breaking the privacy. It provides the proxies with the shares of purposefully designed values. It also performs calculations on the data masked by the proxies and returns the share of the results of these calculations. In our solution, we use fixed-point arithmetic to be able to represent and work on real numbers. A detailed explanation of the number format as well as the algorithms of the complex building blocks can be found in the supplemental information.

Addition

The proxies add the shares of two secret-shared values they have to obtain the share of the addition of these values without any communication or privacy leakage.

Multiplication

The multiplication operation, which we adapted from SecureML, uses the precomputed multiplication triples[41] and requires truncation because of the special number format. For more details, please refer to Wagh et al.[22] and Mohassel and Zhang.[23]

CellPress
OPEN ACCESS

Modulo conversion

We offer the functionality MOC converting shares over $\mathbb{Z}_K$ to fresh shares over $\mathbb{Z}_L$, where $L = 2K$. Even though other frameworks in the literature have functions with a similar name, none of them perform this specific MOC. SecureNN,[22] for instance, offers *ShareConvert* to convert the shares from 2^{64} to $2^{64} - 1$. Assuming that P_0 and P_1 have the shares $\langle x \rangle_0^K$ and $\langle x \rangle_1^K$, respectively, the first step for P_0 and P_1 is to mask their shares by using the shares of the random value $r \in \mathbb{Z}_K$ sent by P_2. Afterward, they reconstruct $(x + r) \in \mathbb{Z}_K$ by first computing $\langle y \rangle_i^K = \langle x \rangle_i^K + \langle r \rangle_i^K$ for $i \in \{0, 1\}$ and then sending these values to each other. Along with the shares of $r \in \mathbb{Z}_K$, P_2 also sends the information in Boolean shares telling whether the summation of the shares of r wraps so that P_0 and P_1 can convert r from the ring $\mathbb{Z}_K$ to the ring $\mathbb{Z}_L$. Once they reconstruct $y \in \mathbb{Z}_K$, P_0 and P_1 can change the ring of y to $\mathbb{Z}_L$ by adding K to one of the shares of y if $\langle y \rangle_0^K + \langle y \rangle_1^K$ wraps. After conversion, the important detail regarding $y \in \mathbb{Z}_L$ is to fix the value of it. P_0 and P_1 identify if $(x + r) \in \mathbb{Z}_K$ wraps using the private compare (PC) method,[22] and then P_0, P_1, or both add K to their shares depending on the Boolean share of the outcome of PC. If both add, then this means that there is no addition to the value of $y \in \mathbb{Z}_L$. At the end, P_i subtracts $r_i \in \mathbb{Z}_L$ from $y_i \in \mathbb{Z}_L$ and obtains $x_i \in \mathbb{Z}_L$ for $i \in \{0,1\}$.

Most significant bit

One of the biggest improvements that we introduce is in the private determination of the MSB of a secret-shared value x via MSB. We deftly integrated MOC and PC into MSB so that we could significantly reduce the communication round complexity of it. Given the shares of x, MSB first extracts the least significant $(n - 1)$-bits via mod K. Then, it converts the ring of this value from $\mathbb{Z}_K$ to $\mathbb{Z}_L$ and subtracts it from x. This results in either 0 or K in $\mathbb{Z}_L$, and MSB secretly maps it to 0 or 1, respectively, to obtain the MSB of x.

Comparison

We also provide CMP, which privately compares two secret-shared values, x and y, and outputs 1 if $x > y$, and 0 otherwise, in secret-shared form. CMP utilizes MSB to determine the MSB of $(x - y)$ and return the output of MSB as the output of CMP.

Multiplexer

We address the private selection via the functionality MUX. It performs the selection of one of two secret-shared values based on the secret-shared selection bit value using the RE of multiplication.[42] In MUX, the proxies compute the following using their shares $(\langle x \rangle_0, \langle y \rangle_0, \langle b \rangle_0)$ and $(\langle x \rangle_1, \langle y \rangle_1, \langle b \rangle_1)$:

$$z = x - b(x - y) = \langle x \rangle_0 + \langle x \rangle_1 - \langle b \rangle_0(\langle x \rangle_0 - \langle y \rangle_0) - \langle b \rangle_1(\langle x \rangle_1 - \langle y \rangle_1) - \langle b \rangle_0(\langle x \rangle_1 - \langle y \rangle_1) - \langle b \rangle_1(\langle x \rangle_0 - \langle y \rangle_0) \quad \text{(Equation 1)}$$

Then, they obtain the fresh shares of $z = x - b(x - y)$. As shown in Equation 1, the proxies need to multiply two values owned by different parties in the computation of $\langle b \rangle_0(\langle x \rangle_1 - \langle y \rangle_1)$ and $\langle b \rangle_1(\langle x \rangle_0 - \langle y \rangle_0$. They outsource these multiplications to the helper via the RE. They first prepare six components of the encoding of this function using a set of random values and send four of them to the helper party. The helper party then combines these components in a way that results in a partially decrypted intermediate result. It secret shares this intermediate result to the proxies, and, as the final step, the proxies subtract their share of the intermediate result from the unsent component of the encoding to obtain $\langle b \rangle_0(\langle x \rangle_1 - \langle y \rangle_1)$, $\langle b \rangle_1(\langle x \rangle_0 - \langle y \rangle_0$, and, eventually, the selected value privately. This application of RE demonstrates its potential as a tool for secure MPC protocol design.

Matrix product

Since the matrix product is a widely used operation in machine learning algorithms, we provide MP. It essentially uses the same idea of MUL, and we use the same optimization as Wagh et al.[22] Please refer to that study for further details. Note that we perform dot product operations represented as DP in Figure 2 using MP too.

Exponential

Even though the private EXP has been addressed before,[43,44] it is inefficient and/or approximates the fractional and negative EXP. In this study, we introduce the exact exponential functionality *EXP*. It computes the exact exponential of a publicly known base raised to the power of a given secret-shared value, which has been computed by approximation so far in the literature. For this purpose, we have been inspired by the square-and-multiply algorithm and extended the core idea of the square-and-multiply algorithm to cover the exact exponential computation of not only the positive numbers but also the negative numbers as well as their decimal parts in a multi-party scenario. As an overview, the proxies first obtain the MSB of the secret-shared power and use this to select the set containing either the power itself and the contribution of each bit of a positive power or the absolute of the power and the contribution of each bit of a negative power. Then, the proxies determine the value of each bit of the power in a secret-sharing form and use them to select between the previously selected contributions of the bits and a vector of 1s. The last step is to multiply these selected contributions of the bits of the power to the exponential in a binary-tree-like structure. In total, *EXP* requires two MSB, two MUX, and $log_2(n)$-many MUL. Our exponential can also be extended to address the EXP when the base is also secret shared.

Security analysis

Lemma 1. The protocol MOC securely realizes the functionality $\mathcal{F}_{MOC}$ in the $\mathcal{F}_{PC}$ hybrid model

Proof. First, we prove the correctness of our protocol by showing $(\langle x \rangle_0^K + \langle x \rangle_1^K) \bmod K = (\langle x \rangle_0 + \langle x \rangle_1) \bmod L$. In the protocol, $y = (x + r) \bmod K$ and isWrap$(x, r, K) = r \overset{?}{>} y$, that is, isWrap$(x, r, K) = 1$ if $r > y$ and 0 otherwise. At the beginning, P_0,P_1, and P_2 call $\mathcal{F}_{PC}$ to compute $c = r \overset{?}{>} y$, and P_0 and P_1 obtain the Boolean shares c_0 and c_1, respectively. Besides, P_2 also sends the Boolean shares w_0 and w_1 of $w = \text{isWrap}(\langle r \rangle_0, \langle r \rangle_1, K)$ to P_0 and P_1, respectively. If isWrap$(\langle y \rangle_0, \langle y \rangle_1, K)$ is 1, then P_0 adds K to $\langle y \rangle_0$ to change the ring of y from K to L. To convert r from ring K to ring L, P_0 and P_1 add K to their shares of r based on their Boolean shares w_0 and w_1, respectively. If $w_0 = 1$, then P_0 adds K to its r_1 and P_1 does the same with its shares. Later, we need to fix is the summation of x and r, that is, the value y. In the case of $x + r \geq K$, we cannot fix the summation value y in ring L by simply converting it from ring K to ring L. This summation should be $x + r$ in ring L rather than $(x + r)$ mod K. To handle this problem, P_0 and P_1 add K to their shares of y based on their shares c_0 and c_1. As a result, we convert the values y and r to ring L and fix the value of y if necessary. The final step to obtain x_i for party P_i is to simply subtract r_i from y_i where $i \in \{0,1\}$.

Next, we prove the security of our protocol. P_2 involves this protocol in execution of $\mathcal{F}_{PC}$. We give the proof $\mathcal{F}_{PC}$ above. At the end of the execution of $\mathcal{F}_{PC}$, P_2 learns u'. However, $u' = u \oplus (x > r)$, and P_2 does not know u. Thus, u' is uniformly distributed and can be perfectly simulated with randomly generated values. P_i where $i \in \{0, 1\}$ sees fresh shares of $\langle r \rangle_i^K$, $\{\langle r[j] \rangle_i^V\}_{j \in [n]}$, w_i^B, and u_i^B. These values can be perfectly simulated with randomly generated values.

Lemma 2. The protocol MSB securely realizes the functionality $\mathcal{F}_{MSB}$ in the $\mathcal{F}_{MOC}$ hybrid model

Proof. First, we prove the correctness of our protocol. Assume that we have n-bit number u. $v = u - (u mod 2^{n-1})$ is either 0 or 2^{n-1}. In our protocol, $\langle z \rangle_i$ is the output of P_i where $i \in \{0, 1\}$. We have to prove that Reconstruct$(\langle z \rangle_i)$ is equal to the MSB of x. P_i where $i \in \{0, 1\}$ computes $d_i^K = x_i \bmod K$, which is a share of d over K. P_i computes d_i, which is a share of d over L by invoking MOC. Note that $z = x -$ Reconstruct$(\langle d \rangle_i)$, and all bits of z are 0 except the MSB of z, which is equal to the MSB of x. Now, we have to map z to 1 if it is equal to K or 0 if it is equal to 0. P_0 sends the z_0 and $z_0 + K$ in random order to P_2, and P_1 sends the z_1 to P_2. P_2 reconstructs two different values, divides these values by K, creates two additive shares of them, and sends these shares to P_0 and P_1. Since P_0 and P_1 know the order of the real MSB value, they correctly select the shares of its mapped value.

Second, we prove the security of our protocol. P_i where $i \in \{0, 1\}$ sees $\langle d \rangle_i$, which is a fresh share of d, and $\langle a[0] \rangle_i$ and $\langle a[1] \rangle_i$, one of which is a fresh share of the MSB of x and the other is a fresh share of the complement of the MSB of x. Thus, the view of P_i can be perfectly simulated with randomly generated values.

Lemma 3. The protocol CMP securely realizes the functionality $\mathcal{F}_{CMP}$ in the $\mathcal{F}_{MSB}$ hybrid model

Proof. First, we prove the correctness of our protocol. Assume that we have x and y. We first compute $z = y - x$. If z is negative, which corresponds to 1 in the MSB of z, then it means that $x > y$. In this case, CMP outputs 1. If z is non-negative, which corresponds to 0 in the MSB of z, then it indicates that $y \geq x$. In this case, the output of CMP is 0. Since the output of CMP exactly matches the

output of MSB and we have already proved the correctness of MSB, we can conclude that CMP works correctly.

Second, we prove the security of our protocol. Since $\langle z\rangle_i = \langle y\rangle_i - \langle x\rangle_i$ is computed locally by P_i for $i \in \{0,1\}$, it does not reveal any information about x and y. Afterward, MSB is called on $\langle z\rangle_i$ to determine the MSB of z in secret-shared form. Considering that the security of MSB is proven, we can conclude that CMP compares two secret-shared values without compromising their privacy.

Lemma 4. The protocol MUX securely realizes the functionality $\mathcal{F}_{MUX}$

Proof

We first prove the correctness of our protocol. $\langle z\rangle_i$ is the output of P_i where $i \in \{0,1\}$. We need to prove that Reconstruct$(\langle z\rangle_i) = (1-b)x + by$.

$$\begin{aligned}\langle z\rangle_0 + \langle z\rangle_1 = {} & \langle x\rangle_0 - \langle b\rangle_0(\langle x\rangle_0 - \langle y\rangle_0) + r_1\langle b\rangle_0 + r_2(\langle x\rangle_0 - \langle y\rangle_0) + \\ & r_2r_3 + \langle x\rangle_1 - \langle b\rangle_1(\langle x\rangle_1 - \langle y\rangle_1) + r_0(\langle x\rangle_1 - \langle y\rangle_1) + \\ & r_0r_1 + r_3\langle b\rangle_1 - \langle b\rangle_0\langle x\rangle_1 + \langle b\rangle_0\langle y\rangle_1 - \langle b\rangle_0 r_1 - r_0\langle x\rangle_1 + \\ & r_0\langle y\rangle_1 - r_0r_1 - \langle x\rangle_0\langle b\rangle_1 - \langle x\rangle_0 r_2 + \langle y\rangle_0\langle b\rangle_1 + \langle y\rangle_0 r_2 - \\ & r_3\langle b\rangle_1 - r_3r_2 = (1 - \langle b\rangle_0 - \langle b\rangle_1)(\langle x\rangle_0 + \langle x\rangle_1) + \\ & (\langle b\rangle_0 + \langle b\rangle_1)(\langle y\rangle_0 + \langle y\rangle_1) = (1 - b)x + by\end{aligned} \quad \text{(Equation 2)}$$

Next, we prove the security of our protocol. P_2 gets M_2, M_3, M_5, and M_6. All these values are uniformly random values because they are generated using uniformly random values r_0, r_1, r_2, and r_4. P_2 computes $M_2M_5 + M_3M_6$. The computed value is still uniformly random because it contains uniformly random values r_0, r_1, r_2, and r_4. As a result, any value learned by P_2 is perfectly simulated. For each $i \in \{0,1\}$, P_i learns a fresh share of the output. Thus, P_i cannot associate the share of the output with the shares of the inputs, and any value learned by P_i is perfectly simulatable.

Lemma 5. The protocol* EXP *securely computes the exponential of a publicly known base raised to the power of a secret-shared value

Proof. We begin the proof by showing the correctness of the method. Let x be the power whose representation in our number format is $\langle x\rangle$ and b be the publicly known base. P_0 or P_1 computes $C_p = \{\ldots, b^8, b^4, b^2, b, b^{1/2}, b^{1/4}, b^{1/8}, \ldots\}$ and $C_n = \{\ldots, b^{-8}, b^{-4}, b^{-2}, b^{-1}, b^{-1/2}, b^{-1/4}, b^{-1/8}, \ldots\}$, and the other generates a corresponding set of 0s for C_p and C_n. These values in C_p and C_n correspond to b^{2^i} and $b^{-1 \cdot 2^i}$, respectively, for $i \in \{(n-f), \ldots, 2, 1, 0, -1, -2, \ldots, -f\}$, assuming that only the corresponding bit value of the power x is 1. They choose one of these sets based on the sign of x and let C be the selected set. Afterward, they must choose between $c_j \in C$ and 1 depending on $\langle x\rangle_j$ where $j \in \{0, 1, \ldots, n\}$. For this selection, they use the MSB operation on all cases where each bit of x is at the MSB position. This is done by shifting the shares of x to the left. Once they have the correct set of contributions, they basically multiply all of those contributions to obtain the result of the exponential. This proves the correctness of EXP.

Corruption of a proxy

At the beginning, since the adversary corrupting a proxy knows only one share of the power x, that is, either x_0 or x_1, it cannot infer any information about the other share. The first step of the exponential is to compute the possible contribution of every bit of positive and negative power. This is publicly known. The following step is to select between these contributions depending on the result of MSB(x) by using MUX. Since both MSB and MUX are secure, the adversary can neither infer anything about x_{1-j} nor relate the share of the result it obtains to x in general. In the next step, they obtain each bit of x in secret-shared form by using MSB and bit shifting on the shares of x. Considering the proven security of MSB and the shifting being simply local multiplication of each share by 2, there is no information that the adversary could obtain. Afterward, the proxies select the correct contributions by employing MUX. Since MUX gives the fresh share of what is selected, the adversary cannot associate the inputs with the output. The last step is to multiply these selected contributions via MUL, which is also proven to be secure. Therefore, we can conclude that *EXP* is secure against a semi-honest adversary corrupting a proxy.

Corruption of the helper

Since the task of the helper party in the computation of the exponential of a secret-shared power is to either provide multiplication triples or perform the required computation on the masked data, there is nothing that the adversary corrupting the helper party could learn about x. Therefore, it is fair to state that *EXP* is secure against a semi-honest adversary corrupting the helper.

SUPPLEMENTAL INFORMATION

Supplemental information can be found online at https://doi.org/10.1016/j.patter.2024.101023.

ACKNOWLEDGMENTS

This study is supported by the DFG Cluster of Excellence "Machine Learning – New Perspectives for Science," EXC 2064/1, project number 390727645, and the German Ministry of Research and Education (BMBF), project number 01ZZ2010.

AUTHOR CONTRIBUTIONS

A.B.U., N.P., and M.A. contributed to the idea development, experiment design, result evaluation, and paper writing. A.B.U. and M.A. designed and implemented the building blocks, and they did the security analyses of these building blocks. A.B.U. conducted the experiments and plotted the figures.

DECLARATION OF INTERESTS

The authors declare no competing interests.

Received: December 12, 2023
Revised: February 17, 2024
Accepted: June 13, 2024
Published: July 19, 2024

REFERENCES

1. Kallel, A., Rekik, M., and Khemakhem, M. (2022). Hybrid-based framework for covid-19 prediction via federated machine learning models. J. Supercomput. *78*, 7078–7105.
2. Qin, H., Zawad, S., Zhou, Y., Padhi, S., Yang, L., and Yan, F. (2020). Reinforcement-learning-empowered mlaas scheduling for serving intelligent internet of things. IEEE Internet Things J. *7*, 6325–6337.
3. Alabbadi, M.M. (2011). Mobile learning (mlearning) based on cloud computing: mlearning as a service (mlaas). Proc. UBICOMM, 296–302.
4. Anfinsen, C.B. (1973). Principles that govern the folding of protein chains. Science *181*, 223–230.
5. Orengo, C.A., Todd, A.E., and Thornton, J.M. (1999). From protein structure to function. Curr. Opin. Struct. Biol. *9*, 374–382.
6. Chen, K., Kurgan, L.A., and Ruan, J. (2008). Prediction of protein structural class using novel evolutionary collocation-based sequence representation. J. Comput. Chem. *29*, 1596–1604.
7. Gohlke, H., and Klebe, G. (2002). Approaches to the description and prediction of the binding affinity of small-molecule ligands to macromolecular receptors. Angew. Chem. Int. Ed. *41*, 2644–2676.
8. Yang, Y., Faraggi, E., Zhao, H., and Zhou, Y. (2011). Improving protein fold recognition and template-based modeling by employing probabilistic-based matching between predicted one-dimensional structural properties of query and corresponding native properties of templates. Bioinformatics *27*, 2076–2082.
9. Liu, B., Li, C.-C., and Yan, K. (2020). Deepsvm-fold: protein fold recognition by combining support vector machines and pairwise sequence similarity scores generated by deep learning networks. Briefings Bioinf. *21*, 1733–1741.
10. Zhang, C., Zheng, W., Mortuza, S., Li, Y., and Zhang, Y. (2020). Deepmsa: constructing deep multiple sequence alignment to improve contact prediction and fold-recognition for distant-homology proteins. Bioinformatics *36*, 2105–2112.
11. Jumper, J., Evans, R., Pritzel, A., Green, T., Figurnov, M., Ronneberger, O., Tunyasuvunakool, K., Bates, R., Žídek, A., Potapenko, A., et al. (2021). Highly accurate protein structure prediction with alphafold. Nature *596*, 583–589.

12. Varadi, M., Anyango, S., Deshpande, M., Nair, S., Natassia, C., Yordanova, G., Yuan, D., Stroe, O., Wood, G., Laydon, A., et al. (2022). Alphafold protein structure database: massively expanding the structural coverage of protein-sequence space with high-accuracy models. Nucleic acids research *50*, D439–D444.
13. Lin, Z., Akin, H., Rao, R., Hie, B., Zhu, Z., Lu, W., dos Santos Costa, A., Fazel-Zarandi, M., Sercu, T., Candido, S., et al. (2022). Language models of protein sequences at the scale of evolution enable accurate structure prediction. Preprint at bioRxiv. https://doi.org/10.1101/2022.07.20.500902.
14. Chen, D., Jacob, L., and Mairal, J. (2019). Recurrent kernel networks. Adv. Neural Inf. Process. Syst. *32*.
15. Abadi, M., Chu, A., Goodfellow, I., McMahan, H.B., Mironov, I., Talwar, K., and Zhang, L. (2016). Deep learning with differential privacy. In Proceedings of the 2016 ACM SIGSAC conference on computer and communications security, pp. 308–318.
16. Chen, S., Fu, A., Shen, J., Yu, S., Wang, H., and Sun, H. (2020). Rnn-dp: A new differential privacy scheme base on recurrent neural network for dynamic trajectory privacy protection. J. Netw. Comput. Appl. *168*, 102736.
17. Bakshi, M., and Last, M. (2020). Cryptornn-privacy-preserving recurrent neural networks using homomorphic encryption. In International Symposium on Cyber Security Cryptography and Machine Learning (Springer), pp. 245–253.
18. Hesamifard, E., Takabi, H., and Ghasemi, M. (2017). Cryptodl: Deep neural networks over encrypted data. Preprint at arXiv. https://doi.org/10.48550/arXiv.1711.05189.
19. Gilad-Bachrach, R., Dowlin, N., Laine, K., Lauter, K., Naehrig, M., and Wernsing, J. (2016). Cryptonets: Applying neural networks to encrypted data with high throughput and accuracy. In International conference on machine learning (PMLR), pp. 201–210.
20. Lu, W.-j., Huang, Z., Hong, C., Ma, Y., and Qu, H. (2021). Pegasus: bridging polynomial and non-polynomial evaluations in homomorphic encryption. In 2021 IEEE Symposium on Security and Privacy (SP) (IEEE), pp. 1057–1073.
21. Knott, B., Venkataraman, S., Hannun, A., Sengupta, S., Ibrahim, M., and van der Maaten, L. (2021). Crypten: Secure multi-party computation meets machine learning. Adv. Neural Inf. Process. Syst. *34*, 4961–4973.
22. Wagh, S., Gupta, D., and Chandran, N. (2019). SecureNN: 3-Party Secure Computation for Neural Network Training. Proc. Priv. Enhancing Technol. *2019*, 26–49.
23. Mohassel, P., and Zhang, Y. (2017). SecureML: A System for Scalable Privacy-Preserving Machine Learning. In 2017 IEEE symposium on security and privacy (SP) (IEEE), pp. 19–38.
24. Damgård, I., Pastro, V., Smart, N., and Zakarias, S. (2012). Multiparty computation from somewhat homomorphic encryption. In Annual Cryptology Conference (Springer), pp. 643–662.
25. Rathee, D., Rathee, M., Kumar, N., Chandran, N., Gupta, D., Rastogi, A., and Sharma, R. (2020). Cryptflow2: Practical 2-party secure inference. In Proceedings of the 2020 ACM SIGSAC Conference on Computer and Communications Security, pp. 325–342.
26. Wagh, S., Tople, S., Benhamouda, F., Kushilevitz, E., Mittal, P., and Rabin, T. (2020). Falcon: Honest-majority maliciously secure framework for private deep learning. Preprint at arXiv. https://doi.org/10.48550/arXiv.2004.02229.
27. Patra, A., Schneider, T., Suresh, A., and Yalame, H. (2021). {ABY2. 0}: Improved {Mixed-Protocol} secure {Two-Party} computation. In 30th USENIX Security Symposium (USENIX Security 21), pp. 2165–2182.
28. Mishra, P., Lehmkuhl, R., Srinivasan, A., Zheng, W., and Popa, R.A. (2020). Delphi: A cryptographic inference service for neural networks. In 29th USENIX Security Symposium (USENIX Security 20), pp. 2505–2522.
29. Huang, Z., Lu, W.-j., Hong, C., and Ding, J. (2022). Cheetah: Lean and fast secure two-party deep neural network inference. IACR Cryptol. ePrint Arch. *2022*, 207.
30. EL-Manzalawy, Y., Dobbs, D., and Honavar, V. (2008). Predicting linear b-cell epitopes using string kernels. J. Mol. Recogn.: An Interdisciplinary Journal *21*, 243–255.
31. Nojoomi, S., and Koehl, P. (2017). A weighted string kernel for protein fold recognition. BMC Bioinf. *18*, 1–14.
32. Leslie, C., Eskin, E., and Noble, W.S. (2001). The spectrum kernel: A string kernel for svm protein classification. In Biocomputing 2002 (World Scientific), pp. 564–575.
33. Hochreiter, S., Heusel, M., and Obermayer, K. (2007). Fast model-based protein homology detection without alignment. Bioinformatics *23*, 1728–1736.
34. Dobson, C.M. (2001). The structural basis of protein folding and its links with human disease. Philosophical Transactions of the Royal Society of London. Series B: Biological Sciences *356*, 133–145.
35. Wang, Z., and Moult, J. (2001). Snps, protein structure, and disease. Hum. Mutat. *17*, 263–270.
36. Yue, P., Li, Z., and Moult, J. (2005). Loss of protein structure stability as a major causative factor in monogenic disease. Journal of molecular biology *353*, 459–473.
37. Lieberman, R.L. (2019). How does a protein's structure spell the difference between health and disease? our journey to understand glaucoma-associated myocilin. PLoS Biol. *17*, e3000237.
38. Nielsen, J.B., Nordholt, P.S., Orlandi, C., and Burra, S.S. (2012). A new approach to practical active-secure two-party computation. In Annual Cryptology Conference (Springer), pp. 681–700.
39. Murzin, A.G., Brenner, S.E., Hubbard, T., and Chothia, C. (1995). Scop: a structural classification of proteins database for the investigation of sequences and structures. Journal of molecular biology *247*, 536–540.
40. Ünal, A.B. (2024). mdppml/RKN-as-a-Service: Source code of "A Privacy Preserving Approach for Cloud-Based Protein Fold Recognition. Zenodo. https://doi.org/10.5281/zenodo.11546407.
41. Beaver, D. (1991). Efficient Multiparty Protocols Using Circuit Randomization. In Advances in Cryptology - CRYPTO '91, 11th Annual International Cryptology Conference, Santa Barbara, California, USA, August 11-15, 1991, Proceedings, pp. 420–432. https://doi.org/10.1007/3-540-46766-1_34.
42. Applebaum, B. (2017). Garbled circuits as randomized encodings of functions: a primer. In Tutorials on the Foundations of Cryptography (Springer), pp. 1–44.
43. Keller, M., and Sun, K. (2022). Secure quantized training for deep learning. In International Conference on Machine Learning (PMLR), pp. 10912–10938.
44. Aly, A., and Smart, N.P. (2019). Benchmarking privacy preserving scientific operations. In Applied Cryptography and Network Security: 17th International Conference, ACNS 2019, Bogota, Colombia, June 5–7, 2019, Proceedings (Springer), pp. 509–529.

Patterns, Volume *5*

Supplemental information

A privacy-preserving approach for cloud-based protein fold recognition

Ali Burak Ünal, Nico Pfeifer, and Mete Akgün

Supplementary Note 1: Fixed-point Arithmetic

Since machine learning algorithms can require both positive and negative real numbers, we use fixed-point arithmetic to represent numbers. In this number format, the most significant bit is allocated for the sign of the value. 0 represents positive and 1 represents negative values. Besides the most significant bit, a certain number of the least significant bits of this representation are used for the fractional part of the value and the rest expresses the integer part.

To illustrate the number format, let n be the number of bits to represent numbers, f be the number of bits allocated for the fractional part and $\mathbb{S}$ be the set of values that can be represented in this number format, one can convert $x \in \mathbb{R}$ to $\hat{x} \in \mathbb{S}$ as follows:

$$\hat{x} = \begin{cases} \lfloor x * 2^f \rfloor & x \geq 0 \\ 2^n - \lfloor |x * 2^f| \rfloor & x < 0 \end{cases} \tag{1}$$

For example, $x = 3.42$ is represented as $\hat{x} = 112066$, which, if we omit the leading zeros, is 1101101011100001010001 in binary for $f = 20$. The two most significant bits, the 21st and 22nd bits, are used to represent 3 and the remainder represents 0.42.

The choice of f depends on the required precision by the task. If the numbers in the task are mostly small or they need to be as accurate as possible, the precision of the representation of the numbers is crucial. In such a case, a higher value for f is required to allow for more decimal places of the original value, which in return requires sacrifices in the upper limit that can be calculated by the building blocks. However, if the numbers that appear during the process are large, f must be set to lower values in order to allow the integer part to represent larger numbers with fewer decimal places.

Supplementary Note 2: Algorithms

1 **Algorithm** MOC()
  input : P_0 and P_1 hold $\langle x \rangle_0^K$ and $\langle x \rangle_1^K$, respectively
  output: P_0 and P_1 get $\langle x \rangle_0$ and $\langle x \rangle_1$, respectively
2 P_0 and P_1 hold a common random bit u'
3 P_2 picks a random number $r \in \mathbb{Z}_K$ and generates $\langle r \rangle_0^K$, $\langle r \rangle_1^K$, $\{\langle r[j] \rangle_0^V\}_{j \in [n]}$ and $\{\langle r[j] \rangle_1^V\}_{j \in [n]}$.
4 P_2 computes $w =$ isWrap$(\langle r \rangle_0^K, \langle r \rangle_1^K, K)$ and divides w into two boolean shares w_0^B and w_1^B
5 P_2 sends $\langle r \rangle_i^K$, $\{\langle r[j] \rangle_i^V\}_{j \in [n]}$ and w_i^B to P_i, for each $i \in \{0, 1\}$
6 For each $i \in \{0, 1\}$, P_i executes Steps 7-8
7 $\langle y \rangle_i^K = \langle x \rangle_i^K + \langle r \rangle_i^K$
8 P_i reconstructs y by exchanging shares with P_{1-i}
9 $u_i^B =$ PC$(\{\langle r[j] \rangle_i^V\}_{j \in [n]}, y, u')$
10 P_0 computes $u_i^B = u_i^B \oplus u'$
11 For each $i \in \{0, 1\}$, P_i computes $c_i^B = w_i^B \oplus u_i^B$
12 P_0 computes $\langle y \rangle_0 = \langle y \rangle_0^K +$ isWrap$(\langle y \rangle_0^K, \langle y \rangle_1^K, K) \cdot K$
13 P_1 sets $\langle y \rangle_1 = \langle y \rangle_1^K$
14 For each $i \in \{0, 1\}$, P_i computes $\langle x \rangle_i = \langle y \rangle_i - (\langle r \rangle_i^K + c_i^B \cdot K)$

Algorithm 1: Modulus Conversion (MOC)

1 **Algorithm** MSB()

input : P_0 and P_1 hold $\langle x \rangle_0$ and $\langle x \rangle_1$, respectively

output: P_0 and P_1 get $\langle z \rangle_0$ and $\langle z \rangle_1$, respectively, where z is equal to 0 if the most significant bit of x is 0 and 1 otherwise.

2 P_0 and P_1 hold a common random bit f and g. P_0 and P_1 additionally hold ℓ common random values $s_j \in \mathbb{Z}_V^*$ for all $j \in [\ell]$, a random permutation π for ℓ elements and ℓ common random values $u_j \in \mathbb{Z}_V^*$.

3 P_2 picks a random number $r \in K$ and generates $\langle r \rangle_0^K$, $\langle r \rangle_1^K$, $\{\langle r[j] \rangle_0^V\}_{j \in [\ell]}$ and $\{\langle r[j] \rangle_1^V\}_{j \in [\ell]}$.

4 P_2 computes $w =$ isWrap$(\langle r \rangle_0^K, \langle r \rangle_1^K, K)$

5 P_2 sends $\langle r \rangle_i^K$ and $\{\langle r[j] \rangle_i^V\}_{j \in [\ell]}$ to P_i, for each $i \in \{0, 1\}$

6 For each $i \in \{0, 1\}$, P_i executes Steps 7-26

7 $\langle d \rangle_i^K = \langle x \rangle_i \mod K$

8 $\langle y \rangle_i^K = \langle d \rangle_i^K + \langle r \rangle_i^K$

9 $y =$ Reconst$(\langle \mathrm{y} \rangle_\mathrm{i}^\mathrm{K})$

10 $\langle y \rangle_i = \langle y \rangle_i^K + i \cdot$ isWrap$(\langle y \rangle_0^K, \langle y \rangle_1^K, K) \cdot K$

11 $\langle a[0] \rangle_i = ifK - \langle x \rangle_i + \langle y \rangle_i - \langle r \rangle_i^K$

12 $\langle a[1] \rangle_i = i(1-f)K - \langle x \rangle_i + \langle y \rangle_i - \langle r \rangle_i^K$

13 Let $t = y + 1 \bmod 2^\ell$

14 **for** $j = \ell - 1;\ j > 0;\ j = j - 1$ **do**

15   **if** $g = 0$ **then**

16     $\langle w_j \rangle_i^V = \langle r[j] \rangle_i^V + iy[j] - 2y[j]\langle r[j] \rangle_i^V$

17     $\langle c_j \rangle_i^V = iy[j] - \langle r[j] \rangle_i^V + j + \sum_{k=j+1}^{\ell} \langle w_k \rangle_i^V$

18   **else if** $g = 1$ *AND* $r \neq 2^\ell - 1$ **then**

19     $\langle w_j \rangle_i^V = \langle r[j] \rangle_i^V + it[j] - 2t[j]\langle r[j] \rangle_i^V$

20     $\langle c_j \rangle_i^V = -it[j] + \langle r[j] \rangle_i^V + i + \sum_{k=j+1}^{\ell} \langle w_k \rangle_i^V$;

21   **else**

22   **if** $i \neq 1$ **then**

23       $\langle c_j \rangle_i^V = (1-i)(u_j + 1) - iu_j$

24   **else**

25       $\langle c_j \rangle_i^V = (-1)^j \cdot u_j$

26 Send $\left\{\langle b_j \rangle_i^V\right\}_j = \pi\left(\left\{s_j \langle c_j \rangle_i^V\right\}_j\right)$ and $\langle a \rangle_i$ to P_2

27 For all $j \in [\ell]$, P_2 computes $d_j =$ Reconst$(\langle d_j \rangle_0^V, \langle d_j \rangle_1^V)$ and sets $g' = 1$ iff $\exists j \in [\ell]$ such that $d_j = 0$.

28 P_2 reconstructs $a[j]$ where $j \in \{0, 1\}$ and computes $a[j] = (a[j] - (g' \oplus w)K)/K$

29 P_2 creates two fresh shares of $a[j]$ where $j \in \{0, 1\}$ and sends them to P_0 and P_1

30 For each $i \in \{0, 1\}$, P_i executes Step 31

31 $\langle z \rangle_i = \langle a[f \oplus g] \rangle_i$

Algorithm 2: Most Significant Bit (MSB)

Supplementary Note 3: Round Complexity and Execution Time Analysis

We give the round complexity of our building blocks and their runtime on different network settings for specific sizes of input data. Even though the purpose of the paper is not to propose a new MPC framework, we compare our building blocks to similar ones in the literature to demonstrate their efficiency. Considering the similarity of the number of parties, the design of these parties, and the secret sharing technique, SecureNN[1] seems the best choice for such a pur-

1 **Algorithm** MUX()

input : P_0 and P_1 hold ($\langle x\rangle_0$, $\langle y\rangle_0$, $\langle b\rangle_0$) and ($\langle x\rangle_1$, $\langle y\rangle_1$, $\langle b\rangle_1$), respectively.

output: P_0 and P_1 get $\langle z\rangle_0$ and $\langle z\rangle_1$, respectively, where $z = x - b(x - y)$.

2 P_0 and P_1 hold four common random values r_i where $i \in \{0, 1, 2, 3\}$

3 P_0 computes $M_1 = \langle x\rangle_0 - \langle b\rangle_0(\langle x\rangle_0 - \langle y\rangle_0) + r_1\langle b\rangle_0 + r_2(\langle x\rangle_0 - \langle y\rangle_0) + r_2r_3$, $M_2 = \langle b\rangle_0 + r_0$, $M_3 = \langle x\rangle_0 - \langle y\rangle_0 + r_3$

4 P_0 sends M_2 and M_3 to P_2

5 P_1 computes $M_4 = \langle x\rangle_1 - \langle b\rangle_1(\langle x\rangle_1 - \langle y\rangle_1) + r_0(\langle x\rangle_1 - \langle y\rangle_1) + r_0r_1 + r_3\langle b\rangle_1$, $M_5 = (\langle x\rangle_1 - \langle y\rangle_1) + r_1$, $M_6 = \langle b\rangle_1 + r_2$

6 P_1 sends M_5 and M_6 to P_2

7 P_2 computes $M_2M_5 + M_3M_6 = z$

8 P_2 divides z into two shares ($\langle z\rangle_0 + \langle z\rangle_1$) and sends $\langle z\rangle_0$ and $\langle z\rangle_1$ to P_0 and P_1, respectively

9 P_0 computes $\langle z\rangle_0 = M_1 - \langle z\rangle_0$

10 P_1 computes $\langle z\rangle_1 = M_4 - \langle z\rangle_1$

Algorithm 3: Multiplexer (MUX)

1 **Algorithm** EXP()

input : P_0 and P_1 hold $\langle x\rangle_0$ and $\langle x\rangle_1$, respectively, and publicly known base b

output: P_0 and P_1 get $\langle z\rangle_0$ and $\langle z\rangle_1$, respectively

2 For $i \in \{0, 1\}$, P_i executes Steps 3-15 with the help of P_2

3 $\langle s\rangle_i =$ MSB($\langle x\rangle_i$)

4 $\langle |x|\rangle_i = 0 - \langle x\rangle_i$

5 **for** $j = n;\ j > 0;\ j = j - 1$ **do**

6 $\quad\langle cP[n-j]\rangle_i = i * b^{2^{j-f}}$

7 $\quad\langle cN[n-j]\rangle_i = i * (1/b^{2^{j-f}})$

8 $(\langle \hat{x}\rangle_i, \langle cONE\rangle_i) =$ MUX$((\langle x\rangle_i, \langle cP\rangle_i), (\langle |x|\rangle_i, \langle cN\rangle_i), \langle s\rangle_i)$

9 **for** $j = 0;\ j < n;\ j = j + 1$ **do**

10 $\quad\langle \hat{x}^{n-j}\rangle_i = (\langle \hat{x}\rangle_i << j)$

11 $\langle M\rangle_i =$ MSB$((\langle \hat{x}^{64}\rangle_i, \langle \hat{x}^{63}\rangle_i, \ldots, \langle \hat{x}^{1}\rangle_i))$

12 $\langle cACT\rangle_i =$ MUX$(\langle cONE\rangle_i, \langle cZERO\rangle_i, \langle M\rangle_i)$

13 $\langle z\rangle_i = \langle cACT\rangle_i$

14 **for** $j = 0;\ j < log_2(n);\ j = j + 1$ **do**

15 $\quad\langle z\rangle_i =$ MUL$(\langle z[0 : len(z)/2]\rangle_i, \langle z[len(z)/2 : len(z)]\rangle_i)$

Algorithm 4: Exponential computation (EXP)

pose. In our MSB, for instance, we need 4 rounds whereas it requires 5 rounds in SecureNN. Besides, MSB of SecureNN takes the input from the ring $L - 1$ and outputs in the ring L where $L = 2^{64}$. Such inconsistency between the input and the output increases the round complexities of some operations relying on MSB in SecureNN. For example, we can compare the DReLU in SecureNN with our CMP method. We did not include the implementation of the DReLU as it is not necessary for RKN inference. However, DReLU can be considered as the inverse or complement of the CMP process. In DReLU(x), the output is 0 if x is less than 0, and 1 otherwise. On the other hand, CMP(x, y) outputs 1 if $(y - x)$ is less than 0, and 0 otherwise. The calculation of $(y - x)$ is done locally without the need for any communication between the computing parties. The complement of bits in arithmetic secret sharing can be achieved through the subtraction operation, which can be performed locally without requiring communication between the parties. It is evident that these two operations are similar in terms of communication cost and require the same underlying functions. In SecureNN, the DReLU function invokes ShareConvert, which

converts shares from the ring L to the ring $L-1$ in 4 communication rounds. This conversion is necessary because the subsequent function MSB expects inputs in the ring $L-1$, while the inputs to DReLU are in the ring L. As a result, DReLU in SecureNN requires 8 communication rounds, which is 1 less than the summation of the round complexities of $ShareConvert$ and MSB thanks to an optimization that they did while combining. On the other hand, our CMP function only invokes our MSB function, which takes inputs and gives outputs in the ring L without requiring share conversion. Therefore, our CMP only requires 4 communication rounds. **(Table S1)** summarizes the communication round complexities of the utilized building blocks.

It is important to note that even though we have some methods with similar names to the methods in SecureNN, they, in fact, have completely different functionalities. For instance, our MOC method involves converting shares from the 2^{63} ring to the 2^{64} ring. In SecureNN, there is no equivalent method to MOC. The closest method in SecureNN is ShareConvert, which converts shares from the 2^{64} ring to the $(2^{64})-1$ ring. Although both methods require 4 rounds of communication, they differ significantly in terms of input and methodology.

Protocol	RC	LAN Time (ms)	WAN Time (ms)	
			20 ms delay	58 ms delay
MUL	2	7.9	25.6	64.1
MUX	2	6.53	48.2	127
MOC	4	307	377	537
MSB	4	65.6	133	264
CMP	4	68.1	145	264
EXP	12 + 2 $\log n$	536	839	1380

Table S1: Round complexities (RC) of our building blocks where n is the number of bits to represent values. In addition to the round complexity, we also give the runtime of our building blocks on different network settings for input vectors of size 10^5.

References

1. Wagh, S., Gupta, D., and Chandran, N. (2019). SecureNN: 3-Party Secure Computation for Neural Network Training. Proc. Priv. Enhancing Technol. *2019*, 26–49.
